# Ral GEF with the PH Domain and SH3 Binding Motif 1 Regulated by Splicing Factor Junction Plakoglobin and Pyrimidine Metabolism Are Prognostic in Uterine Carcinosarcoma

**DOI:** 10.1155/2021/1484227

**Published:** 2021-10-28

**Authors:** Hongjun Guo, Siqiao Wang, Aiqing Xie, Wenhuizi Sun, Chenlu Wei, Shuyuan Xian, Huabin Yin, Mingxiao Li, Hanlin Sun, Hong Li, Tong Meng, Jie Zhang, Zongqiang Huang

**Affiliations:** ^1^Department of Obstetrics and Gynecology, The First Affiliated Hospital of Zhengzhou University, 1 East Jianshe Road, Zhengzhou, China; ^2^Division of Spine, Department of Orthopedics, Tongji Hospital Affiliated to Tongji University School of Medicine, 389 Xincun Road, Shanghai, China; ^3^Tongji University School of Medicine, 1239 Siping Road, Shanghai 200092, China; ^4^School of Ocean and Earth Science, Tongji University, 1239 Siping Road, Shanghai 200092, China; ^5^Department of Gynaecology, Tongji Hospital Affiliated to Tongji University School of Medicine, 389 Xincun Road, Shanghai, China; ^6^Department of Orthopedics, Shanghai General Hospital, School of Medicine, Shanghai Jiaotong University, 100 Haining Road, Shanghai, China; ^7^Tongji University Cancer Center, Shanghai Tenth People's Hospital, Tongji University School of Medicine, 301 Yanchang Road, Shanghai 200072, China; ^8^Department of Orthopedics, The First Affiliated Hospital of Zhengzhou University, 1 East Jianshe Road, Zhengzhou, China

## Abstract

Uterine carcinosarcoma (UCS) is a highly invasive malignant tumor that originated from the uterine epithelium. Many studies suggested that the abnormal changes of alternative splicing (AS) of pre-mRNA are related to the occurrence and metastasis of the tumor. This study investigates the mechanism of alternative splicing events (ASEs) in the tumorigenesis and metastasis of UCS. RNA-seq of UCS samples and alternative splicing event (ASE) data of UCS samples were downloaded from The Cancer Genome Atlas (TCGA) and TCGASpliceSeq databases, several times. Firstly, we performed the Cox regression analysis to identify the overall survival-related alternative splicing events (OSRASEs). Secondly, a multivariate model was applied to approach the prognostic values of the risk score. Afterwards, a coexpressed network between splicing factors (SFs) and OSRASEs was constructed. In order to explore the relationship between the potential prognostic signaling pathways and OSRASEs, we fabricated a network between these pathways and OSRASEs. Finally, validations from multidimension platforms were used to explain the results unambiguously. 1,040 OSRASEs were identified by Cox regression. Then, 6 OSRASEs were incorporated in a multivariable model by Lasso regression. The area under the curve (AUC) of the receiver operator characteristic (ROC) curve was 0.957. The risk score rendered from the multivariate model was corroborated to be an independent prognostic factor (*P* < 0.001). In the network of SFs and ASEs, junction plakoglobin (JUP) noteworthily regulated RALGPS1-87608-AT (*P* < 0.001, *R* = 0.455). Additionally, RALGPS1-87608-AT (*P* = 0.006) showed a prominent relationship with distant metastasis. KEGG pathways related to prognosis of UCS were selected by gene set variation analysis (GSVA). The pyrimidine metabolism (*P* < 0.001, *R* = −0.470) was the key pathway coexpressed with RALGPS1. We considered that aberrant JUP significantly regulated RALGPS1-87608-AT and the pyrimidine metabolism pathway might play a significant part in the metastasis and prognosis of UCS.

## 1. Introduction

Uterine carcinosarcoma (UCS) is an aggressive variant of endometrial carcinoma characterized by unusual histologic features, including discrete malignant epithelial and mesenchymal components (carcinoma and sarcoma) [1]. Most of the managements for UCS had been extrapolated from researches of endometrial carcinomas and sarcomas [2]. It is a kind of hyperplasia with high invasiveness and distant metastasis of endometrial carcinoma, accounting for about 3% of endometrial cancer.

The prognosis of uterine carcinosarcoma is not optimistic, the 5-year survival rate is estimated to be only 30%, and the mortality rate is 16% of endometrial cancer. In terms of treatment, surgery is the primary treatment for uterine carcinosarcoma (UCS) [3]. Trastuzumab is supposed to be effective for HER2-positive uterine carcinosarcoma patients, whereas anti-HER2 therapy in other gynecological malignancy does not have enough evidence to be efficacious which is under evaluation [4]. Chemotherapy with carboplatin-paclitaxel has no significant effect on the progression survival rate of patients with UCS [5]. Early diagnoses for malignant tumors are essential for the overall survival (OS) of patients. Although confined to the corpus, the recurrence rate of UCS still remains very high, the development and recurrence of which might aggravate the tumor and lead to a poor prognosis [6]. Several studies explored potential prognosis-related genes of UCS [6, 7], and a novel study classified UCS into different subtypes with distinct molecular and clinicopathologic features to improve subtype-specific therapeutic regimens [8]. Nevertheless, the complicated heterogeneity and low frequency of UCS indicate that related researches are insufficient and further study on the pathogenesis of UCS and exploration of novel biomarkers for the improvement of the prognostic prediction of patients with UCS are urgently required. In this context, the role of AS in distant metastasis and prognosis of UCS was explored in this study, which will not only aid in the interpretations of invasion and metastasis mechanisms but in the amelioration of individualized therapeutic methods for UCS.

At present, the researches of UCS mainly focus on the level of gene transcription events and their posttranscription processes and mechanisms have not got enough attention [6, 9]. Genes are transcribed to form precursor mRNAs, which are then alternatively spliced to transform into mature mRNAs. And it leads to the formation of different mRNA subtypes, which are then translated into different proteins [10].

The splicing factors (SFs) dominate the alternative splicing events (ASEs) in these processes, thus constructing complex regulatory networks, leading to the complex and diverse expression products. Cell differentiation, tissue-specific acquisition, and genealogy are closely related to these mechanisms [11].

Abnormal alternative splicing events of some genes lead to the disorder of regulatory networks and the dedifferentiation of somatic cells. It reports that this may cause cell malignant transformation and carcinosarcoma formation [12]. Therefore, the discovery of regulation networks may be helpful to find molecular markers for UCS, so as to find new therapeutic methods and thus improve the prognosis and survival time of patients with UCS.

In this study, to identify overall survival-related ASEs (OSRASEs) of UCS, we comprehensively analyzed AS profiling. On this basis, we built a prognosis prediction model. Significant SFs and ASEs related to metastasis of uterine carcinosarcoma were determined using Pearson analysis, which revealed the possible mechanisms of metastasis of UCS. In addition, we also found feasible targets for UCS metastasis.

## 2. Materials and Methods

### 2.1. Data Collection

Firstly, we collected RNA transcription data, clinical information, and SFs of UCS samples from TCGA Data Portal (https://tcga-data.nci.nih.gov/tcga/) [13]. Then, we downloaded alternative splicing events (ASEs) from the TCGASpliceSeq database (https://bioinformatics.mdanderson.org/TCGASpliceSeq/) [14]. There are seven types of ASEs, including the alternative promoter (AP), exon skip (ES), alternative acceptor site (AA), mutex exon (ME), alternative terminator (AT), reserved intron (RI), and alternative donor site (AD) [15]. Samples were excluded if their percent-spliced-in (PSI) value > 25%. Through data collation and ID conversion, the result matrices of ASEs were composed of the ID number, gene name, and alternative splicing type. Clinical data included the survival time, survival state, age, gender, grade stage, and TMN classification of cancer.

### 2.2. Identification of the OSRASEs

Due to the undetected sample information in the data, we applied the K-nearest neighbor algorithm to minimize the bias. Samples of ASEs with standard deviations < 0.01 were excluded, as well as samples with no follow-up information. Univariate Cox regression analysis was performed to assess prognosis correlation and the value of every sample by integrating clinical data and ASEs. ASE with a *P* value < 0.05 is related to the survival of UCS patients. The UpSet plot was formed to explicate ASEs related to survival and OSRASEs, and the volcano plot was developed to explain the ASEs which were related or unrelated to the prognosis of UCS. The bubble plots were constructed to show expression levels of the top 20 OSRASEs for each type of ASE. Specifically, in bubble plots, the size and color represented different prognostic values of ASEs.

### 2.3. Establishment of the Prognostic Model Related to OSRASEs

Lasso regression analysis was applied to exclude the prognostic factors with high correlation and picked out the top 20 important prognostic OSRASEs, preventing overfitting of the prognostic model. The multivariate Cox regression model was constructed for evaluation of prognostic OSRASEs with high correlation with prognosis, which symbolized the coefficient of correlation of every OSRASE of this model.

Based on the median risk score, the cases were separated into the high-risk group and the low-risk group. In order to evaluate the accuracy of the prognosis model, we drew a ROC curve and calculated the area under it. We also performed the Kaplan-Meier survival analysis to verify the significance of the difference between the low-risk group and the high-risk group. The risk scores were achieved using the formula as follows:

(1) $$\begin{array}{c} \text{Risk score}=\overset{n}{\underset{i=1}{\sum }}\beta n\times \text{PSI}n. \end{array}$$

On the basis of the order of the risk score from low to high, the samples were sorted and risk graphs were generated to evaluate prognosis, as well as the expression heat map and scatter plot. Then, we conducted single-/multifactor independent prognostic analysis to develop two forest maps to assess the significance in the prognosis of the risk score, along with the gender, age, clinical stage, grade, and TNM classification.

### 2.4. Construction of the Correlation and Interaction Network

404 splicing factors (SFs) were obtained from the SpliceAid2 database [16]. To identify the correlation and interaction between OSRASEs and SFs, we performed Pearson correlation analysis. The regulatory network of OSRASEs and SFs was constructed using Cytoscape (3.7.1) [17]. Significant regulatory links (∣correlation coefficient | <0.400 and *P* > 0.001) were extracted to construct this network. OSRASEs and SFs were illustrated as ellipses and arrows separately in the network, in which negative and positive regulations were expressed as green and red lines, respectively, and low- and high-risk levels of OSRASEs were defined as purple and red colors, respectively.

### 2.5. Identification of Stage- and/or Metastasis-Correlated OSRASEs

Revealed by beeswarm plots, the Kruskal-Wallis test and Mann-Whitney-Wilcoxon test were manipulated for the identification of the stage- and/or metastasis-related OSRASEs. Then, we constructed a network to expound on regulatory relationships among the OSRASEs related to the TNM stage and/or metastasis.

### 2.6. Coexpression Explication between ASEs and Signaling Pathways

Aforementioned nonparametric tests were performed to evaluate the correlations between the UCS status and OSRASEs. Beeswarm plots were applied to elaborate upon the significance of these correlations.

Picked out by gene set variation analysis (GSVA) initially [18], the signaling pathways which were highly correlated to prognosis were then analyzed and picked off by performing the univariate Cox analysis. To determine potential downstream functional mechanisms of key OSRASEs, we combined KEGG pathways which were related to prognosis and OSRASEs and then performed the coexpression analysis.

### 2.7. Multidimensional Online Validation

To further validate the relationship between OSRASEs and clinical outcomes of patients with UCS and reduce the bias caused by pure silico analysis, we performed external validation based on other multidimensional online databases. Firstly, by utilizing Pathway Card (https://pathcards.genecards.org/), 6 key genes that were closely related to the selected KEGG pathways were extracted for further analysis. PROGgeneV2 [19], UCSC Xena [20], UALCAN [21], Gene Expression Profiling Interactive Analysis (GEPIA) [22], LinkedOmics [23], Oncomine [24], and cBioPortal [25] demonstrated the expression levels of key genes at a transcription level in UCS. Then, Genotype-Tissue Expression (GTEx) [26] was utilized to show the expression levels of key genes aforementioned in healthy tissues, and the Human Protein Atlas [27] was used to compare the expression levels of these genes between normal tissues and UCS tissues in the protein level. Furthermore, Cancer Cell Line Encyclopedia (CCLE) [28] was utilized to describe the gene expression levels in the cellular level in UCS. Last but not least, STRING [29] was utilized to construct the interaction network based on SFs, OSRASEs, and the potential pathways in this study.

### 2.8. Immunohistochemistry Analysis

We achieved information and slides of IHC from the Human Protein Atlas (HPA). Two seasoned pathologists identified the immunostaining information on every IHC slide to identify the proportion of RALGPS1-positive cancer cells. Then, we calculated and showed it as histochemistry score (*H*-score). The calculation formula is as follows:

(2) $$\begin{array}{c} H-\text{score}=\overset{n}{\underset{i=1}{\sum }}\text{p}\text{i}\times \left(i+1\right). \end{array}$$

“pi” means the proportion of the cells with relevant intensity and “*i*” points to the intensity score.

### 2.9. Statistics Analysis

All the statistics analyses were carried out by R version 3.5.2 (https://www.r-project.org). For continuous variables, mean ± standard deviation was applied in the normal distribution in descriptive statistics. To regulate the data size of the regulatory network in this study, correlation *P* < 0.001 and ∣coefficient | >0.400 were employed as screening criteria to extract key coexpression patterns between OSRASEs and SFs. We utilized percentages and counts to depict categorical variables. If two-tailed *P* < 0.05, we considered it significant and adopted it.

## 3. Results

### 3.1. Summary of OSRASEs and ASEs

**Figure 1:**
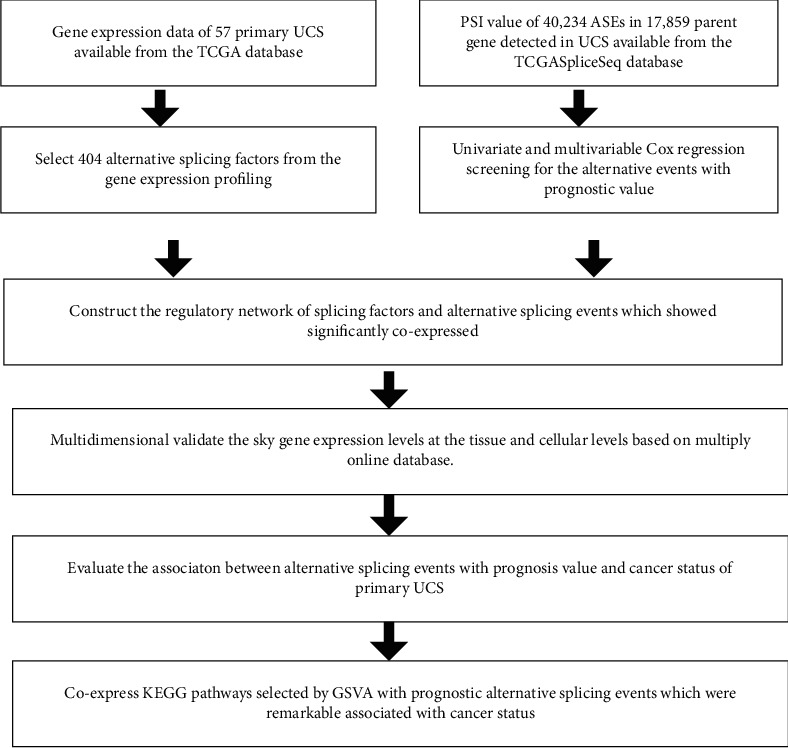
The flowchart of the analysis method.

**Figure 2:**
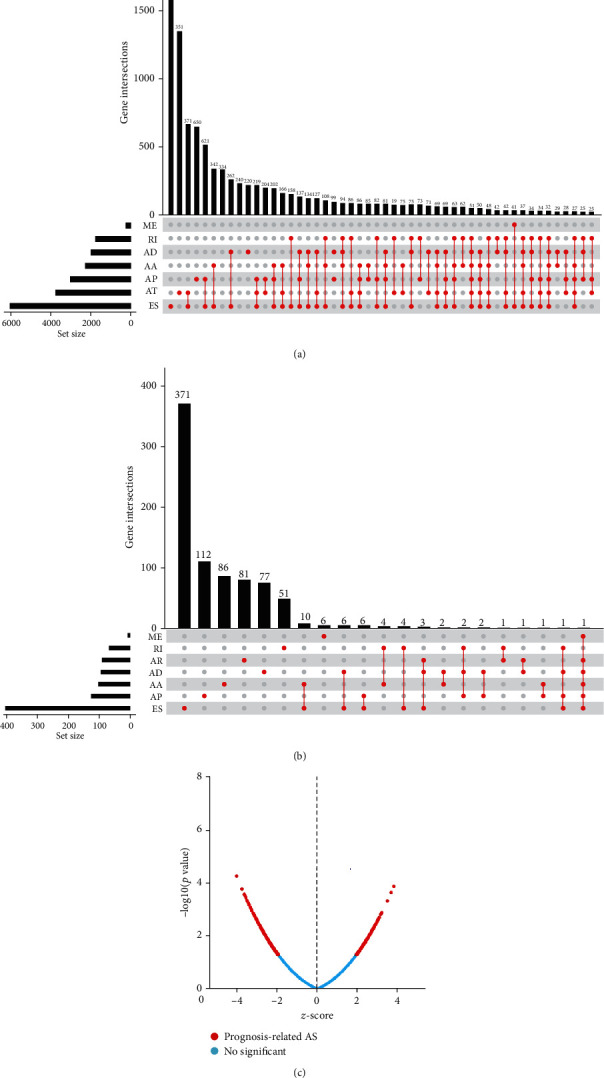
Identification of OSRASEs in UCS patients. The UpSet plots of ASEs and OSRASEs: (a) the number of ASEs in different types of splicing patterns; (b) the number of OSRASEs in different types of splicing patterns; (c) the volcano plot of the prognosis-related and no significant ASEs.

**Figure 3:**
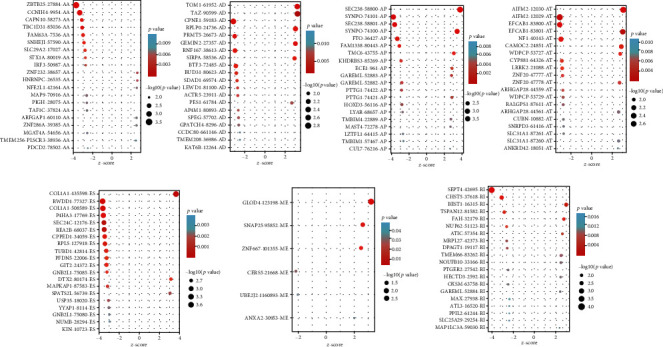
The bubble plots of the top 20 overall survival-associated splicing events, including AA: alternate acceptor site; AD: alternate donor site; AP: alternate promoter; AT: alternate terminator; ES: exon skip; ME: mutex exon; RI: retained intron.

It showed the analysis procedure in Figure 1. Baseline information of 57 patients with UCS was summarized in Table S1. Gene expression data and clinical information of 57 UCS cases were obtained from the TCGA database, and the median survival time was 587 (range, 0–4,269) days. 24 patients died and 10 got tumor metastases in which there were two cases of bone metastasis. A pattern was defined to represent every ASE: the gene name, the TCGASpliceSeq database AS ID of ASE, and splicing pattern were merged as RALGPS1-87608-AT. Specifically, RALGPS1 was the gene name, 87608 was AS ID, and AT was the corresponding splicing pattern. In total, 40,234 ASEs in 17,859 parent genes were discovered in UCS patients which included 3206 AAs in 2,081 genes, 2,817 ADs in 1, 808 genes, 7,612 APs in 2,877 genes, 8, 631 ATs in 3,596 genes, 15,130 ESs in 5,887 genes, 195 MEs in 41 genes, and 2643 RIs in 1,569 genes. Therefore, as can be seen from the figure, a gene could execute more than four alternative splicing patterns (Figure 2(a)). ESs were the most frequent splicing patterns, and the second were ATs. The UpSet plot indicated that ES was the most prevalent alternative splicing pattern-related prognosis of UCS (Figure 2(b)). The Volcano plot demonstrated that the majority of ASEs were OSRASEs (Figure 2(c)). Bubble plots showed the top 20 OSRASEs in the aforementioned 7 alternative splicing patterns (Figures 3(a)–3(g)).

### 3.2. Establishment of the Multivariate Prediction Model

**Figure 4:**
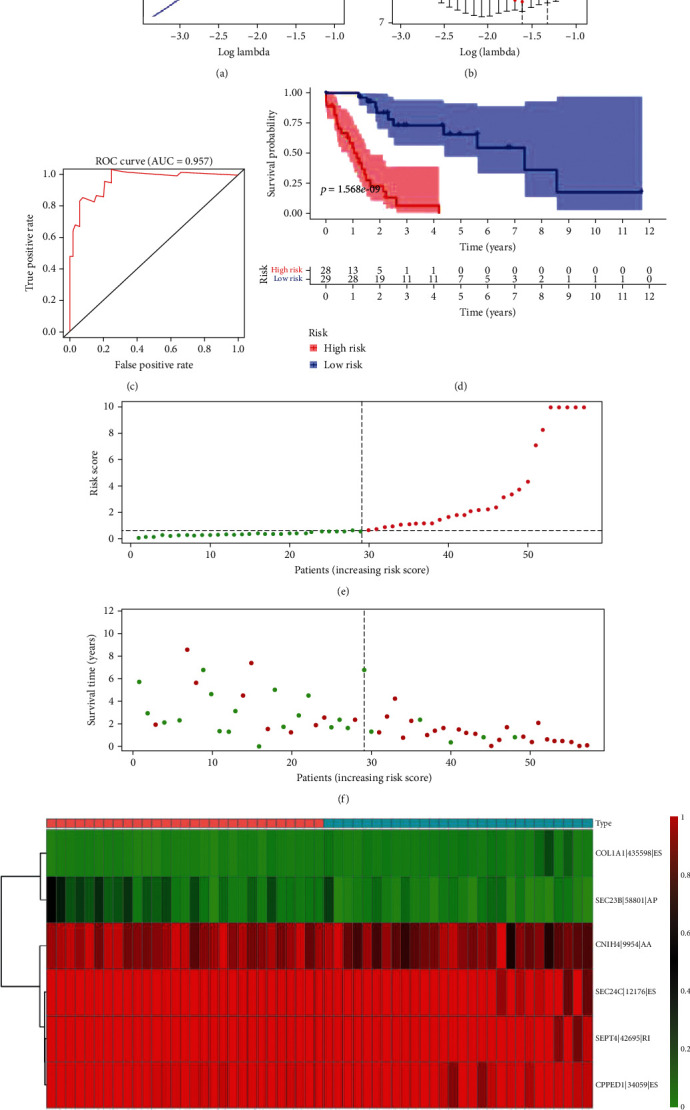
Construction and assessment of the prognosis prediction model. (a) The Lasso regression for top 20 overall survival-associated splicing events with the smallest *P* values, establishment and assessment of the predict model; (b) the coefficients in the Lasso regression for OSRASEs screening; (c) the receiver operator characteristic curve to access the prognosis prediction model (AUC = 0.957); (d) the Kaplan-Meier curve to identify the efficacy of the risk score in overall survival. The high- and low-risk score groups in the (e) scatter plot and (f) risk plot for each sample of UCS based on the profiling from TCGA database; (g) the heat map to illustrate each overall survival-associated splicing event's expression level selected by Lasso regression.

Lasso regression was used to sift the top 20 OSRASEs for preventing overfitting of this prediction model. It indicated that in this multivariate Cox regression analysis, COL1A1-435598-ES, SEC23B-58801-AP, CNIH4-9954-AA, SEC24C-12176-ES, SEPT4-42695-RI, and CPPED1-34059-ES were embraced (Figures 4(a) and 4(b)), with an area under the curve (AUC) of 0.957 (Figure 4(c)). Correspondingly, by calculating the risk score in each case, we obtained a median of 1.056. Further, the Kaplan-Meier plot showed that the prediction model based on the risk score possessed an excellent efficacy (Figure 4(d)).

To elaborate on the relationship between the risk score and vital status of every UCS patient, scatter plot and risk curve were constructed. It can be seen that patients in the low-risk group exhibited a lower mortality compared to those in the high-risk group (Figures 4(e) and 4(f)). The heat map illustrated the expression levels of OSRASEs identified by Lasso regression analysis, in which SEC23B-58801-AP, CNIH4-9954-AA, SEC24C-12176-ES, SEPT4-42695-RI, and CPPED1-34059-ES were lower and COL1A1-435598-ES was higher in the high-risk group (Figure 4(g)).

### 3.3. The Risk Score-Forecasted Prognosis

**Figure 5:**
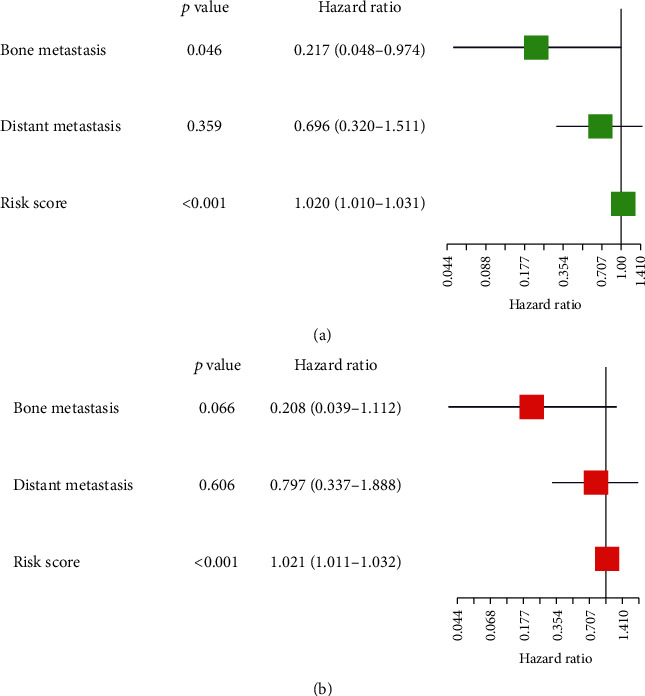
The Cox regression analysis for evaluation of the independent prognostic value of the risk score. (a) Univariate and (b) multivariate cox regression analysis. Forest plots. Green for univariate and red for multivariate.

In the integrated analyses of multivariate and univariate Cox regression, the risk score together with the gender, age, grade, and stage of TMN was appraised. Of all the predictors, the risk score was identified as the most significant and independent one in univariate Cox regression analysis (HR = 1.020, 95% CI (1.010–1.031), *P* < 0.001) (Figure 5(a)), along with multivariate Cox regression analysis (HR = 1.021, 95% CI (1.011–1.032), *P* < 0.001) (Figure 5(b)).

According to the RNA-seq data and relevant clinical information about UCS patients, 390 candidate splicing factors were identified, of which the expression levels were significantly related to the overall survival of patients with UCS.

### 3.4. Construction of the OSRASE and SF Regulatory Network and Metastasis-Related Analysis

**Figure 6:**
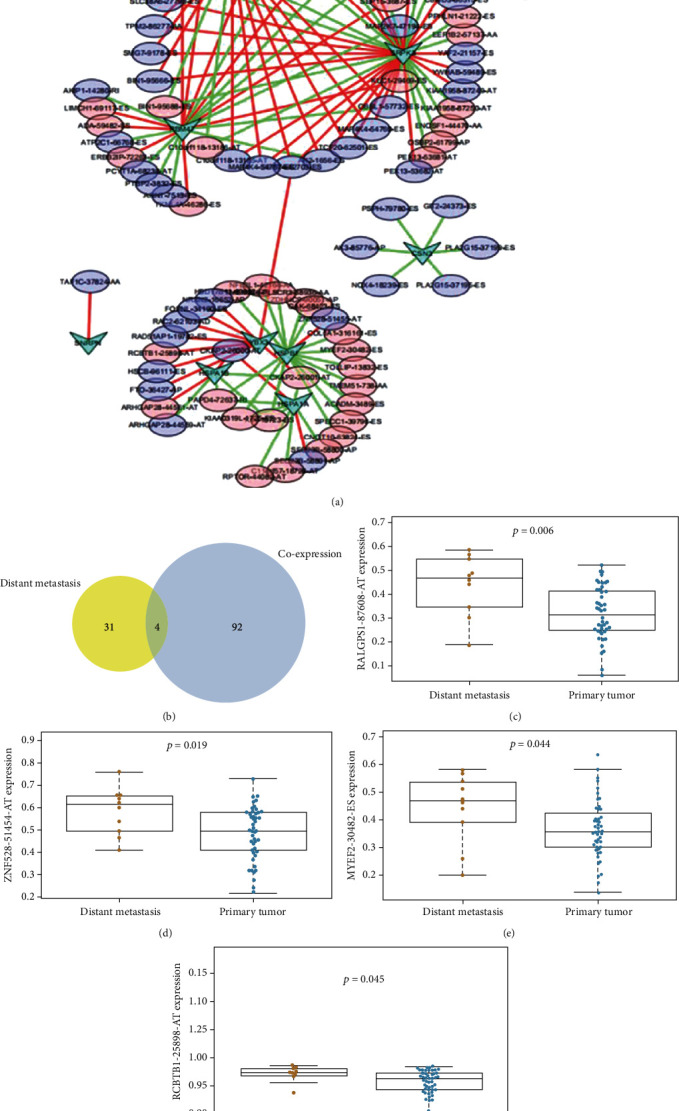
Identification of key metastasis-related OSRASEs. (a) The network constructed for coexpressed splicing factors and overall survival-associated splicing events; arrows represented SFs; the red and blue ellipses represented high and low risks of OSRASEs; (b) the Venn plot showed that there were 35 distant metastasis-associated splicing events and 96 splicing events coexpressed with SFs and their intersections (4 key OSRASEs) were extracted for further analysis; (c) the bar plot to show the relationship between RALGPS1-87608-AT and cancer status; (d) the bar plot to show the relationship between ZNF528-51455-AT and cancer status; (e) the bar plot to show the relationship between MYEF2-30482-ES and cancer status; (f) the bar plot to show the relationship between RCBTB1-25898-AT and cancer status. AT: alternate terminator; ES: exon skip.

To elaborate on the interactions between the SFs and the OSRASEs, we established a regulatory network. In this network, arrows indicated SFs and ellipses indicated OSRASEs with different risk scores. Furthermore, JUP had a significant regulation effect on RALGPS1-87608-AT (*P* < 0.001, *R* = 0.455) in this network (Figure 6(a)).

Among these, in the Venn plot, 4 OSRASEs (RALGPS1-87608-AT, ZNF528-51455-AT, MYEF2-30482-ES, and RCBTB1-25898-AT) were significantly correlated with distant metastasis and coexpressed with SFs (Figure 6(b)). Specifically, RALGPS1-87608-AT was related to metastasis (*P* = 0.006), ZNF528-51454-AT was associated with metastasis (*P* = 0.019), MYEF2-30482-ES was correlated with metastasis (*P* = 0.044), and RCBTB1-25898-AT was related to metastasis (*P* = 0.045) (Figures 6(c)–6(f)).

### 3.5. Coexpression Analysis of Status-Related OSRASEs and Survival-Related Pathways

**Figure 7:**
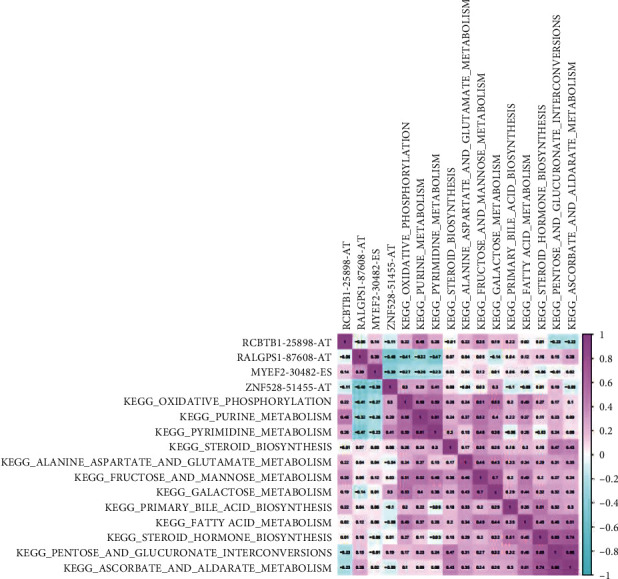
The coexpression analysis between key OSRASEs and signaling pathways. The heat map of coexpression overall survival-associated splicing events related to the cancer status and Kyoto Encyclopedia of Genes and Genomes (KEGG) pathways selected by gene set variation analysis (GSVA).

In order to quantitatively evaluate the enrichment of OSRASEs in different metabolic pathways, we conducted GSVA analysis. By nonparametric and unsupervised analysis, the concentration of OSRASEs in related downstream metabolic pathways was quantified and scored. JUP (SF) was proposed as a remarkable marker associated with RALGPS1-87608-AT (OSRASE) (*P* < 0.001), and the most significantly coexpressed pathways of RALGPS1-87608-AT were pyrimidine metabolism (*P* < 0.001, *R* = −0.470), oxidative phosphorylation (*P* < 0.001, *R* = −0.410), purine metabolism (*P* < 0.001, *R* = −0.320), and ascorbate and aldarate metabolism (*P* < 0.001, *R* = 0.280) (Figure 7).

**Figure 8:**
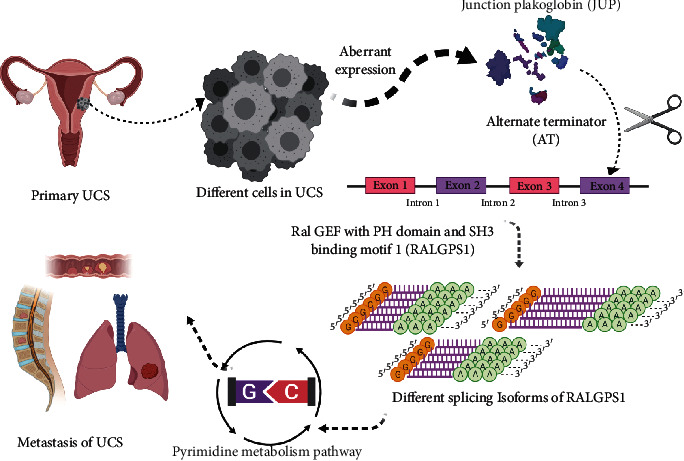
The speculative mechanism diagram including JUP, RALGPS1-87608-AT, and the pyrimidine metabolism pathway.

To sum up, the most significant SF, OSRASEs, and downstream pathway were JUP, RALGPS1-87608-AT, and pyrimidine metabolism, respectively. Finally, the speculative mechanism diagram illustrating the regulatory relationship among JUP, RALGPS1-87608-AT, and the pyrimidine metabolism pathway was summarized in Figure 8.

### 3.6. Multidimensional Validation

To reduce the bias of results in this study, multiomics validation was performed based on various online databases. It showed that ADA, HPRT1, IMPDH1, NUDT2, NUDT9, and PDE4A were the top six genes in pyrimidine metabolism according to the results from Pathway Card. The detail results of multidimensional validation by the Human Protein Atlas (Figure S1), PROGgeneV2 (Figure S2), GEPIA (Figure S3), UCSC Xena (Figure S4), GTEx (Figure S5), UALCAN (Figure S6), LinkedOmics (Figure S7), cBioPortal (Figure S8), Oncomine (Figure S9), CCLE (Figure S10), STRING (Figure S11), and Kaplan-Meier plotter (Figure S12) were summarized in Supplementary Material.

Firstly, expression levels of JUP, RALGPS1, ADA, NUDT9, NUDT2, HPRT1, IMPDH1, and PDE4A in different online platforms were shown in Table S2. NUDT2 was highly expressed, and HPRT1 was less expressed in normal uterine (Figure S1). JUP, ADA, IMPDH1, and HPRT1 were all highly expressed, while RALGPS1, NUDT9, NUDT2, and PDE4A were all less expressed in tumors at the tissue level (Figures S3–S10). RALGPS1, NUDT9, NUDT2, and PDE4A were less expressed in cancer cell lines; JUP, ADA, HPRT1, and IMPDH1 were highly expressed in cancer cell lines in CCLE (Figure S10). A regulatory network of JUP, RALGPS1, ADA, NUDT9, NUDT2, HPRT1, IMPDH1, and PDE4A in STRING was displayed in Figure S11.

Secondly, survival analysis results of JUP, RALGPS1, ADA, NUDT9, NUDT2, HPRT1, IMPDH1, and PDE4A were shown in Table S3. RALGPS1 (*P* = 0.030), NUDT9 (*P* = 0.024), HPRT11 (*P* = 0.004), and PDE4A (*P* = 0.002) were significantly correlated with UCS patients' prognosis in PROGgeneV2 (Figure S2). ADA (*P* = 0.02), IMPDH1 (*P* = 0.03), and PDE4A (*P* < 0.001) were significantly correlated with the clinical stage (Figure S3). And in UALCAN, JUP (*P* = 0.034), ADA (*P* = 0.018), HPRT1(*P* = 0.042), IMPDH1(*P* = 0.019), and PDE4A (*P* < 0.001) were obviously correlated with prognosis (Figure S6). In addition, RALGPS1 (*P* = 0.028), NUDT9 (*P* = 0.024), HPRT1 (*P* = 0.004), IMPDH1 (*P* = 0.040), and PDE4A (*P* = 0.002) were significantly correlated with prognosis in LinkedOmics (Figure S7). Furthermore, JUP (*P* = 0.006), ADA (*P* < 0.001), NUDT9 (*P* < 0.001), NUDT2 (*P* = 0.003), HPRT1 (*P* = 0.030), IMPDH1 (*P* = 0.029), and PDE4A (*P* = 0.048) were significant genes related to the prognosis in the Kaplan-Meier plotter (Figure S12). Finally, Table S4 summarized the results of multidimensional external validation.

## 4. Discussion

UCS is a highly invasive and rare gynecological malignant tumor. Its prevalence is less than 5% of all malignant uterine tumors, but its related deaths account for more than 16% of the deaths caused by uterine malignant tumors [30]. It is a biphasic tumor consisting of malignant epithelium and malignant stroma. Most uterine carcinosarcomas have only one epithelial component; the most common one is poorly differentiated serous adenocarcinoma, which can also be endometrial-like carcinoma, clear cell carcinoma, mucinous carcinoma, squamous cell carcinoma, and undifferentiated tissue type. Squamous cell carcinoma is rare as a single epithelial component. Metastatic UCS deteriorated from primary UCS and possesses a variety of subpopulations with transcription properties, while the molecular mechanisms hidden in UCS tumor occurrence and distant metastasis have not yet been confirmed. Furthermore, available diagnostic and prognostic targets are still in a state of scarcity.

In previous studies, various parameters including the clinical stage, epithelial component grade, performance status, expression level of CA-125, myometrial invasion, adjuvant therapy, and residual cancer were considered to be related to the survival rate of patients with UCS [31]. In recent years, anomalous ASEs related to SFs were identified to be significant in researching cancer biology and clinical treatments as potential factors [10, 32]. ASEs and SFs have been convinced to manufacture various oncoprotein isoforms related to cancer cell proliferation, antiapoptosis, and clinical metastasis [33]. Interestingly, a recent study integrated data of ASEs from the SpliceSeq database and clinical information of HCC from TCGA and a prognostic prediction model based on ORASEs in hepatocellular carcinoma (HCC) was established, providing candidate biomarkers and targets for patients with hepatocellular carcinoma [34]. In addition, another research also constructed a regulation network to elucidate the underlying mechanisms of ORASEs in HCC [35]. Besides, ASEs varied among different cancer types; a previous study identified differential expressing isoforms and ASEs in adenocarcinoma and squamous cell lung cancer cells, providing candidate markers and drug targets for lung cancer [36]. Moreover, a novel research explored the role of ASEs and filled the vacancy of underlying tumorigenesis and metastasis mechanisms of kidney renal clear cell carcinoma [37]. Despite studies regarding prognosis, the relationships among SFs, OSRASEs, and downstream signaling pathways hidden in distant metastasis and prognosis of UCS remained unclear and the ORASE regulatory networks and relative prognostic models for UCS have not yet been clearly determined.

In this study, a total of 1,035 OSRASEs were determined and we established a prognosis predicting model for high-risk population which was based on 4 significant OSRASEs (RCBTB1-25898-AT, RALGPS1-87608-AT, MYEF2-30482-ES, and ZNF528-51455-AT) filtered by Lasso regression analysis. The prediction model in this study had a higher reliability (AUC: 0.957) and fewer predictors, compared with prevenient UCS prediction models. Additionally, we also found that RALGPS1-87608-AT was significantly associated with pyrimidine metabolism, oxidative phosphorylation, purine metabolism, ascorbate, and aldarate metabolism, which were proposed to be the hidden regulation and impressive function of RALGPS1 in distant metastasis of UCS. It was worth mentioning that the risk score was confirmed as an independent prognosis-related factor, predicting remarkable serviceability for patients with UCS.

The junction plakoglobin (JUP) is known to be a desmosomal anchor protein gene, the normal function of which is critical for microtubules and intercellular junctions [38]. It encodes an important cytoplasmic protein, the only known component common to submembranous plaques of intermediate junctions and desmosomes. ASEs occur in its downstream mechanisms. Membrane-related plaques are architectural elements in a critical strategic position to act on the arranging and functional regulation of the cytoskeleton and various cell types. It also plays a significant part in the construction and functional regulation of submembranous plaques, which is also considered as an important tumor suppressor [38]. At the same time, JUP functions as a substrate for VE-PTP and is necessary for it to stimulate VE-cadherin function in endothelial cells. In addition, mutations in JUP and/or changes in its expression levels have been identified in various cancer types (hepatoma, lung adenocarcinoma, and breast cancer) [38–40] and upregulation of it may lead to metastasis and recurrence in patients with squamous cell carcinoma [41]. Thus, we proposed that abnormal expression of JUP might also play an important role in the metastasis and recurrence of UCS. Although there are several experimental researches of JUP regulation both in mice [42, 43], the underlying pathological mechanisms in UCS were revealed, just the tip of the iceberg. Therefore, this study provided a new insight in candidate splicing factors and therapeutic targets for UCS.

Ral GEF with the PH domain and SH3 binding motif 1 (RALGPS1), the parent gene of RALGPS1-87608-AT, was confirmed by various online databases among the identified OSRASEs associated with metastasis and prognosis of patients with UCS. RALGPS1 dysfunction might include abnormalities of biogenesis of GPI dependent on the DPM complex and, with any decrease in THY-1, might yield possible clues in pathophysiology of ovarian teratomas [44]. RALGPS1 belongs to a family of RAS guanine nucleotide exchange factors (GEFs). Activation of it can stimulate signaling pathways which are implicated in the activation a variety of downstream TFs, upregulating the expression levels of other genes associated with cellular division and proliferation. It also corresponds to RALA and RALB, interacting with various downstream effectors and signaling pathways [45]. RALA could combine with many downstream effectors and regulate a variety of cellular activities. As a scaffold and RhoGAP for other proteins, an effector of RALA is RAL-binding protein (RALBP1), which influences receptor-mediated endocytosis actin organization, mitochondrial division, and autophagy [45]. RALBP1 is an important effector for several RAL-driven processes, interacting with various proteins which modulated the endocytosis process and signaling transduction. The Eps homology domain-containing proteins Reps1 and Reps2 were proteins interacting with RALBP1 C-terminus, which were significant for receptor tyrosine kinase-regulated endocytosis [46, 47]. RAL effectors and effector functions play an important role in the occurrence, development, and distant metastasis of numerous tumors especially in UCS.

Another functional protein related to C-terminus of RALBP1 is cyclin B1 [48]. RalBP1 interacts with the activated cyclinB1 enzyme, which is critical for the mitotic phosphorylation of Epsin. Upon phosphorylation, Epsin is no longer available to conduct endocytosis [48]. In addition, the activity was regulated via activation of RALA. Thus, the aberrant function of these factors may cause the formation of cancer cells and lead to UCS.

RALBP1 was identified in screens for proteins, which combine with activated RALA [49–51]. Two ATP-binding motifs in RALBP1 were momentous for transport function. The transport function can promote the export of chemotherapeutic drugs, as well as oxidative damage byproducts induced by radiation therapy [52]. Thus, it shows that RALBP1 is closely linked to the prognosis and efficacy of UCS patients. The overexpression of RALBP1 had been found in various tumors, the inhibition of which can impair tumorigenic growth [53]. This indicated that it may become an effective target for the treatment for UCS.

RALA is identified to recruit RALBP1 to mitochondria, where it plays as a scaffold to stimulate cyclin B phosphorylation and facilitates mitochondrial fission. In addition, mitochondrias can be preserved in each cell equally via a balance of fission and fusion in the process of mitosis. During mitosis, fission promotes equal distribution of mitochondria to daughter cells. Mitochondrial dynamics were identified to be reprogrammed in cancer cells by recruiting mitochondria in cortical cytoskeleton [54]. The mechanisms could enhance the membrane machinery of cellular movements, cellular motility kinase phosphorylation, invasion, chemotaxis, and distant metastasis [54, 55]. At the same time, through GSVA analysis, we found that oxidative phosphorylation, the negative regulated signal pathway of RALGPS1, is closely related to mitochondrial complex I deficiency, mitochondrial complex II deficiency, and mitochondrial complex III deficiency. Therefore, suppression of RALGPS1 may cause mitochondrial fission failure so as to inhibit tumor invasion, chemotaxis, and metastasis. Surprisingly, RALGPS1 was verified to show low expression in UCS patients in our study and various external databases which provided strong evidence for our scientific hypothesis.

Pyrimidine metabolism, a downstream signaling pathway significantly related to JUP and RALGPS1 in this study, is able to encompass various enzymes implicated in synthesis, interconversion, degradation, salvage, and molecule transport [56]. Pyrimidines are important structural components of acids, vitamins, nucleic, nucleotides, folates, and pterins, each one of them fulfilling crucial roles, and disorders of pyrimidine metabolism pathways may cause various malignancies [57]. The pyrimidine metabolism had been widely explored among various organisms. Importantly, critical participation of pyrimidine metabolism in processes which include DNA/RNA synthesis, generation of UDP-sugars for glycosylation of proteins, and generation of precursors activated by CDP made them attractive for study. More importantly, actively dividing cells need more pyrimidines than those in quiescent cells, so tumor cells were identified to overexpress pyrimidine metabolic enzymes universally [58, 59]. ADA catalyzes the hydrolytic deamination of adenosine and 2-deoxyadenosine [60, 61] and plays an important role in purine metabolism and in adenosine homeostasis. Furthermore, it acts as a positive regulator of T-cell coactivation by binding DPP4 [62] and stimulates plasminogen activation [63]. NUDT9 encodes protein which belong to the Nudix hydrolase family, and alternatively spliced transcript variants encoding different isoforms have been found for this gene. NUDT2 asymmetrically hydrolyzes Ap4A to yield AMP and ATP and thus plays a major role in maintaining homeostasis. Interestingly, NUDT2 may be a candidate tumor suppressor gene. Alternative splicing has been observed at this locus, and four transcript variants, all encoding the same protein, have been identified. HPRT1 plays a central role in the generation of purine nucleotides through the purine salvage pathway by which transfers the 5-phosphoribosyl group from 5-phosphoribosylpyrophosphate onto the purine. IMPDH1 catalyzes the conversion of inosine 5′-phosphate (IMP) to xanthosine 5′-phosphate (XMP), the first committed and rate-limiting step in the de novo synthesis of guanine nucleotides, and therefore plays an important role in the regulation of cell growth. It may also play a critical part in the tumorigenesis and progression of several cancer types. PDE4A hydrolyzes the second messenger cAMP, which is a key regulator of many important physiological processes, besides that alternatively spliced transcript variants encoding different isoforms had been described for this gene. Therefore, abnormal metabolism of pyrimidine may play a significant role in the germination, metastasis, and the prognosis of UCS.

Overall, JUP was the key SF and RALGPS1 was the key OSRASE related to the status and distant metastasis of UCS in this study. In addition, pyrimidine metabolism is the potential signaling pathway in the downstream of JUP and RALGPS1.

There were still some deficiencies in the present study. Firstly, the scientific hypothesis in the present study was mainly based on pure bioinformatics analysis, which had not been confirmed by further experiments. Secondly, though the key genes and their regulatory mechanisms were validated using online databases, sequencing data used in this study were obtained from only one cohort with limited sample size. Thirdly, only original data were obtained from TCGA database and the deficiency of samples of metastatic sites, including the bladder, colorectum, and liver, resulted in less integrated results.

To make our hypothesis more reliable and scientific, in the future, a basic experiment will be carried out based on various researches of ASEs, including ASEs in pan-cancer and pancreatic cancer [64, 65]. All genes in our scientific hypothesis will be identified in various samples (caner vs healthy and cancer vs adjacent normal tissue) using IHC for detecting the differential expression. By performing coimmunoprecipitation and RNA immunoprecipitation, a direct mechanism between JUP and RALGPS1 will be confirmed. Moreover, an engineered SF would be applied to identify the ASEs producing specific splicing isoforms of RALGPS1. In addition, immunofluorescence staining will also be applied for validation of the cellular locations of JUP and RALGPS1. The pyrimidine metabolism pathway and distant metastasis of UCS will be further validated using biological function assays such as rescue assays, which can provide more evidence for the potential therapeutic targets and novel prognostic factors in UCS.

In conclusion, we established the prediction model with excellent performance in external database validation. Based on the comprehensive bioinformatics analysis, we considered that aberrant JUP-regulated RALGPS1 might be related to the tumorigenesis, metastasis, and poor prognosis of UCS via pyrimidine metabolism.

## Data Availability

The datasets generated and/or analysed during the current study are available in the in the Supplementary Material and TCGA-UCS program.

## Supplementary Materials

Supplementary Materials Table S1: baseline information of 57 patients with uterine carcinosarcoma available from the TCGA database. Table S2: summary of multidimensional external validation results based on multiple databases. Table S3: summary of the overall survival and stage of multidimensional external validation results based on multiple databases. Table S4: summary of multidimensional external validation. Figure S1: the Human Protein Atlas database validation. (A) JUP was medium expressed in normal uterine tissues and highly expressed in UCS; (B) NUDT9 was moderately expressed in normal uterine tissues and highly expressed in UCS; (C) NUDT2 was highly expressed both in normal uterine tissues and in UCS; (D) the expression of HPRT1 was relatively low in normal tissues compared to that in tumor tissues; (E) IMPDH1 was moderately expressed in normal uterine tissues and less expressed in UCS; (F) IMPDH1 was moderately expressed in normal uterine tissues and less expressed in UCS; (G) PDE4A was not detected in normal uterine tissues and moderately expressed in UCS. Figure S2: PROGgeneV2 database validation. RALGPS1 (B), NUDT9 (D), HPRT1 (F), and PDE4A (H) were significantly correlated with patients' prognosis. Figure S3: GEPIA database validation. RALGPS1 (B), NUDT9 (D), NUDT2 (E), and PDE4A (H) were significantly downregulated in tumor tissues. ADA (K), IMPDH1 (O), and PDE4A (P) were significantly correlated with the clinical stage. Figure S4: UCSC Xena database validation. The expression levels of JUP (A), RALGPS1 (B), NUDT2 (E), HPRT1 (F), and IMPDH1 (G) were relatively highly expressed in early-stage UCS patients compared to advanced UCS patients. ADA (C), NUDT9 (D), and PDE4A (H) were relatively less expressed in early-stage UCS patients compared to advanced UCS patients. Figure S5: GTEx database validation. Heat maps show the expression levels of the genes in different normal tissues. Figure S6: UALCAN database validation. JUP (A), ADA (C), NUDT2 (E), IMPDH1 (G), and PDE4A (H) were all differentially expressed between tumor and normal tissues with significance. Besides, ADA (K), HPRT1 (N), IMPDH1 (O), and PDE4A (P) were significantly correlated with patients' prognosis. Figure S7: LinkedOmics database validation. K-M curves show that high expression of RALGPS1 (B), NUDT9 (D), HPRT1 (F), IMPDH1 (G), and PDE4A (H) was positively related to the negative prognosis in patients with UCS. (I, J) Heat maps show the genes which have noteworthy correlation coefficient with RALGPS1. Figure S8: CBioportal database validation. Expression levels of JUP (A), RALGPS1 (B), ADA (C), NUDT9 (D), NUDT2 (E), HPRT1 (F), IMPDH1 (G), and PDE4A (H) in patients with UCS. (I) Integrated genes (*P* = 0.0007) were significantly related to prognosis. Figure S9: Oncomine database validation. JUP (A), ADA (C), HPRT1 (F), and IMPDH1 (G) were significantly upregulated, while RALGPS1 (B), NUDT9 (D), NUDT2 (E), and PDE4A (H) were downregulated in different kinds of uterine cancers. Figure S10: CCLE database validation. RALGPS1 (B), NUDT9 (D), NUDT2 (E), and PDE4A (H) were downregulated, while JUP (A), ADA (C), HPRT1 (F), and IMPDH1 (G) were significantly upregulated in different kinds of uterine cancers. Figure S11: STRING database validation. The protein-protein interaction network shows that JUP, RALGPS1, ADA, NUDT9, NUDT2, HPRT1, IMPDH1, and PDE4A were closely interacted. Figure S12: K-M Plotter database validation. JUP (A), ADA (C), NUDT9 (D), NUDT2 (E), HPRT1 (F), IMPDH1 (G), and PDE4A (H) were significantly correlated with patients' prognosis.
