# Supplementary material for: Ral GEF with the PH Domain and SH3 Binding Motif 1 Regulated by Splicing Factor Junction Plakoglobin and Pyrimidine Metabolism Are Prognostic in Uterine Carcinosarcoma
Source: Dis Markers. 2021 Oct 28;2021:1484227. doi: 10.1155/2021/1484227 (PMC8568522; doi:10.1155/2021/1484227)
Supplement: Supplementary Materials — Table S1: baseline information of 57 patients with uterine carcinosarcoma available from the TCGA database. Table S2: summary of multidimensional external validation results based on multiple databases. Table S3: summary of the overall survival and stage of multidimensional external validation results based on multiple databases. Table S4: summary of multidimensional external validation. Figure S1: the Human Protein Atlas database validation. (A) JUP was medium expressed in normal uterine tissues and highly expressed in UCS; (B) NUDT9 was moderately expressed in normal uterine tissues and highly expressed in UCS; (C) NUDT2 was highly expressed both in normal uterine tissues and in UCS; (D) the expression of HPRT1 was relatively low in normal tissues compared to that in tumor tissues; (E) IMPDH1 was moderately expressed in normal uterine tissues and less expressed in UCS; (F) IMPDH1 was moderately expressed in normal uterine tissues and less expressed in UCS; (G) PDE4A was not detected in normal uterine tissues and moderately expressed in UCS. Figure S2: PROGgeneV2 database validation. RALGPS1 (B), NUDT9 (D), HPRT1 (F), and PDE4A (H) were significantly correlated with patients' prognosis. Figure S3: GEPIA database validation. RALGPS1 (B), NUDT9 (D), NUDT2 (E), and PDE4A (H) were significantly downregulated in tumor tissues. ADA (K), IMPDH1 (O), and PDE4A (P) were significantly correlated with the clinical stage. Figure S4: UCSC Xena database validation. The expression levels of JUP (A), RALGPS1 (B), NUDT2 (E), HPRT1 (F), and IMPDH1 (G) were relatively highly expressed in early-stage UCS patients compared to advanced UCS patients. ADA (C), NUDT9 (D), and PDE4A (H) were relatively less expressed in early-stage UCS patients compared to advanced UCS patients. Figure S5: GTEx database validation. Heat maps show the expression levels of the genes in different normal tissues. Figure S6: UALCAN database validation. JUP (A), ADA (C), NUDT2 (E), IMPDH1 (G), and PDE4A (H) were [file 1484227.f1.pdf]

Supplementary

Table S1 Baseline information of 57 patients with uterine carcinosarcoma available from the TCGA database.

**Variables**	**Total Patients (N = 57)**
**Age, years**	
Mean ± SD	71.20 ± 10.67
Median	71.00
**Survival time, days**	
Mean ± SD	865.05 ± 851.86
Median	587.00
**Gender**	
Female	57 (100.00%)
**Race**	
Caucasian	44 (77.19%)
African-american	9 (15.79%)
Asian	4 (7.02%)
**Cancer stage**	
Stage 1	22 (38.60%)
Stage 2	5 (8.77%)
Stage 3	20 (35.09%)
Stage 4	10 (17.54%)

SD, Standard deviation.

Table S2. Summary of multidimensional external validation results base on multiple databases

**Database**	**JUP**		**RALGPS1**		**ADA**		**NUDT9**		**NUDT2**		**HPRT1**		**IMPDH1**		**PDE4A**		**Results**
	N	U	N	U	N	U	N	U	N	U	N	U	N	U	N	U	
**The human protein atlas**	-	NA	ND	NA	ND	NA	-	NA	↑	NA	↓	NA	-	NA	ND	NA	NUDT2 was high-expressed, while HPRT1 was low-expressed in normal cerebral cortex in uterine (Figure S1).
**GEPIA**	↓	↑	↑	↓	↓	↑	↑	↓	↑	↓	↓	↑	↓	↑	↑	↓	RALGPS1, NUDT9, NUDT2, and PDE4A expressed highly in normal tissue and lowly in UCS; JUP, ADA, HPRT1, and IMPDH1 expressed lowly in normal uterine and highly in UCS (Figure S3).
**UCSC xena**	NA	↑	NA	↓	NA	↑	NA	↓	NA	↓	NA	↑	NA	↑	NA	↓	RALGPS1, NUDT9, NUDT2, and PDE4A expressed lowly in UCS; JUP, ADA, HPRT1, and IMPDH1 expressed highly in UCS (Figure S4).
**GTEx**	-	NA	-	NA	↓	NA	↑	NA	↑	NA	-	NA	-	NA	↑	NA	NUDT9, NUDT2, and PDE4A was high-expressed, while ADA was low-expressed in normal uterine tissue (Figure S5).
**UALCAN**	NA	↑	NA	-	NA	↑	NA	↓	NA	↓	NA	NA	NA	↑	NA	-	NUDT9 and NUDT2 expressed lowly in UCS; JUP, ADA, and IMPDH1 expressed highly in UCS (Figure S6).

**Linkedomics**	NA	↑	NA	↓	NA	↑	NA	↓	NA	↓	NA	↑	NA	↑	NA	↓	RALGPS1, NUDT9, NUDT2, and PDE4A expressed lowly in UCS; JUP, ADA, HPRT1, and IMPDH1 expressed highly in UCS (Figure S7).
**cBioportal**	NA	↑	NA	↓	NA	↑	NA	↓	NA	↓	NA	↑	NA	↑	NA	↓	RALGPS1, NUDT9, NUDT2, and PDE4A expressed lowly in UCS; JUP, ADA, HPRT1, and IMPDH1 expressed highly in UCS (Figure S8).
**Oncomine**	NA	↑	NA	↓	NA	↑	NA	↓	NA	↓	NA	↑	NA	↑	NA	↓	RALGPS1, NUDT9, NUDT2, and PDE4A expressed lowly in UCS; JUP, ADA, HPRT1, and IMPDH1 expressed highly in UCS (Figure S9).
**CCLE**	NA	↑	NA	↓	NA	↑	NA	↓	NA	↓	NA	↑	NA	↑	NA	↓	RALGPS1, NUDT9, NUDT2, and PDE4A were low-expressed in tumor cell line; JUP, ADA, HPRT1, and IMPDH1 were high-expressed in tumor cell line in CCLE (Figure S10)

Note: “N” was defined as normal; “U” was defined as Uterine carcinosarcoma;“↑” was defined as a significantly high-expressed gene; “↓” was defined as a significantly low-expressed gene; “NA” was defined as “Not available”; “ND” was defined as “Not detached”; “-” was defined as a gene with no significant difference in expression.

Abbreviations: UCS, Uterine Carcinosarcoma; GTEx, Genotype-Tissue Expression; CCLE, Cancer Cell Line Encyclopedia; GEPIA, Gene Expression Profilling Interactive Analysis.

Table S3. Summary of the overall survival and stage of multidimensional external validation results base on multiple databases

**Database**	**JUP (SF)**	**RALGPS1 (ASE gene)**	**ADA (pathway gene)**	**NUDT9 (pathway gene)**	**NUDT2 (pathway gene)**	**HPRT1 (pathway gene)**	**IMPDH1 (pathway gene)**	**PDE4A (pathway gene)**
**UALCAN**	K–M: P=0.034<br>Stage: P=0.037	K–M: P=0.077<br>Stage: P=0.188	K–M: P=0.018<br>Stage: P=0.009	K–M: P=0.07<br>Stage: P=0.219	K–M: P=0.083<br>Stage: P=0.001	K–M: P=0.042<br>Stage: P=0.087	K–M: P=0.019<br>Stage: P=0.0075	K–M: P=0.00017<br>Stage: P=0.003
**LinkedOmics**	K–M: P=0.64	K–M: P=0.028	K–M:P=0.150	K–M: P=0.024	K–M: P=0.093	K–M: P=0.004	K–M: P=0.040	K–M: P=0.0017
**PROGgeneV2**	K–M: P=0.68	K–M: P=0.030	K–M: P=0.16	K–M: P=0.024	K–M: P=0.06	K–M: P=0. 004	K–M: P=0.050	K–M: P=0.002
**GEPIA**	K-M: P=0.28<br>stage: P=0.09	K-M: P=0.2<br>stage: P=0.389	K-M: P=0.03<br>stage: P=0.02	K-M: P=0.06<br>stage: P=0.554	K-M: P=0.024<br>stage: P=0.08	K-M: P=0.009<br>stage: P=0.33	K-M: P=0.024<br>stage: P=0.03	K-M: P=0.00015<br>stage: P=0.0001
**KaplanMeier plotter**	P=0.0057	P=0.2	P=4.6e-08	P=0.0001	P=0.0033	P=0.03	P=0.029	P=0.048

Table S4. Summary of multidimensional external validation

Database	JUP (SF)	RALGPS1 (ASE gene)	ADA (pathway gene)	NUDT9 (pathway gene)	NUDT2 (pathway gene)	HPRT1 (pathway gene)	IMPDH1 (pathway gene)	PDE4A (pathway gene)
**The human protein atlas**	Normal medium	not detected	not detected	normal medium	normal high	normal low	normal medium	not detected
**PROGgeneV2**	K–M: P=0.68	K–M: P=0.030	K–M: P=0.16	K–M: P=0.024	K–M: P=0.06	K–M: P=0. 004	K–M: P=0.050	K–M: P=0.002
**GEPIA**	K-M: P=0.28<br>stage: P=0.09<br>normal low<br>tumor high	K-M: P=0.2<br>stage: P=0.389<br>normal high<br>tumor low	K-M: P=0.03<br>stage: P=0.02<br>normal low<br>tumor high	K-M: P=0.06<br>stage: P=0.554<br>normal high<br>tumor low	K-M: P=0.024<br>stage: P=0.08<br>normal high<br>tumor low	K-M: P=0.009<br>stage: P=0.33<br>normal low<br>tumor high	K-M: P=0.024<br>stage: P=0.03<br>normal low<br>tumor high	K-M: P=0.00015<br>stage: P=0.0001<br>normal high<br>tumor low
**UCSC xena**	early stage high	early stage high	early stage low	early stage low	early stage high	early stage high	early stage high	early stage low
**GTEx**	normal median	normal median	normal low	normal high	normal high	normal medium	normal medium	normal high
**UALCAN**	K–M: P=0.034<br>Stage: P=0.037	K–M: P=0.077<br>Stage: P= 0.188	K–M: P=0.018<br>Stage:P=0.009	K–M: P=0.07<br>Stage: P=0.219	K–M: P=0.083<br>Stage: P=0.001	K–M: P=0.042<br>Stage: P=0.087	K–M: P=0.019<br>Stage: P=0.0075	K–M: P=0.00017<br>Stage: P=0.003
**LinkedOmics**	K–M: P=0.64	K–M: P=0.028	K–M: P=0.15	K–M: P=0.024	K–M: P=0.093	K–M: P=0.004	K–M: P=0.040	K–M: P=0.0017
**cBioportal**	tumor high	tumor low	tumor high	tumor low	tumor low	tumor high	tumor high	tumor low
**Oncomine**	tumor high	tumor low	tumor high	tumor low	tumor low	tumor high	tumor high	tumor low

**CCLE**	tumor high	tumor low	tumor high	tumor low	tumor low	tumor high	tumor high	tumor low
**String**								
**KaplanMeier plotter**	P=0.0057	P=0.2	P=4.6e-08	P=0.0001	P=0.0033	P=0.03	P=0.029	P=0.048

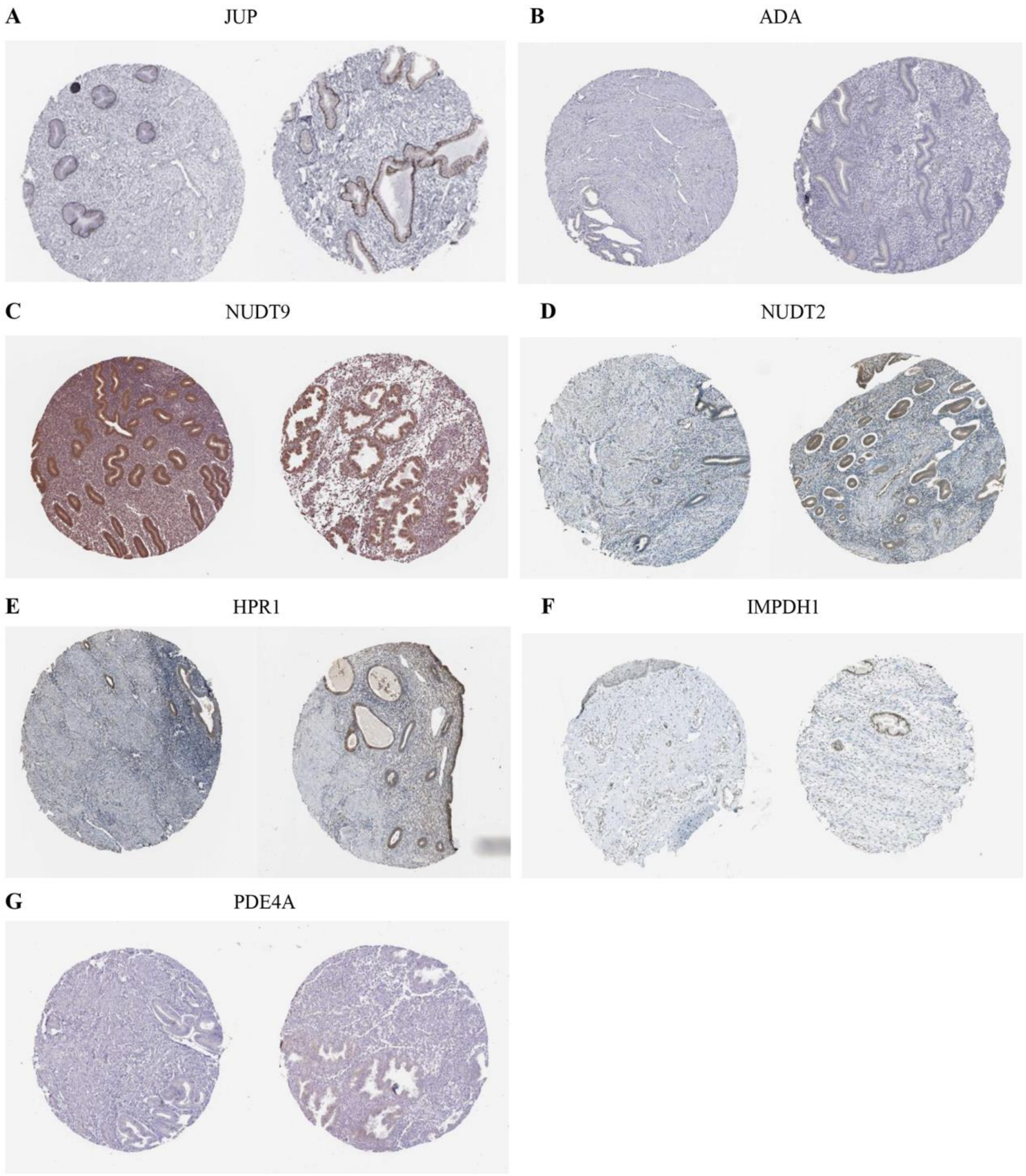

Figure S1 The Human Protein Atlas database validation. (A) JUP was medium expressed in normal uterine tissues and high expressed in UCS; (B) NUDT9 was medium expressed in normal uterine tissues and high expressed in UCS; (C) NUDT2 was high expressed both in normal uterine tissues and UCS; (D) The expression of HPRT1 was relatively low in normal tissues compared to that in tumor tissues; (E) IMPDH1 was medium expressed in normal uterine tissues and low expressed in UCS; (F) IMPDH1 was medium expressed in normal uterine tissues and low expressed in UCS; (G) PDE4A was not detected in normal uterine tissues and medium expressed in UCS.

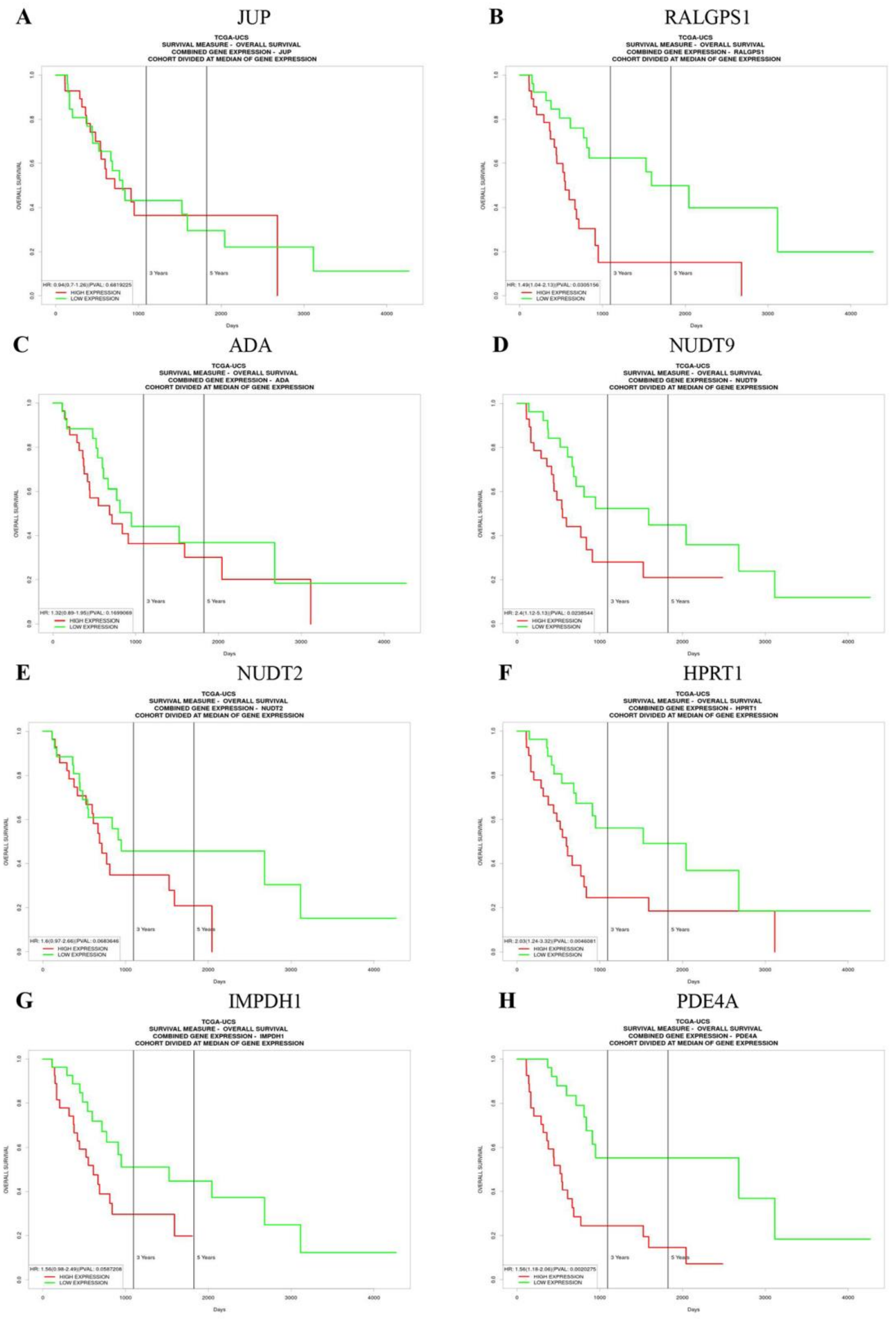

Figure S2 PROGgeneV2 database validation. RALGPS1 (B), NUDT9 (D), HPRT1 (F), and PDE4A (H) were significantly correlated with patients' prognosis.

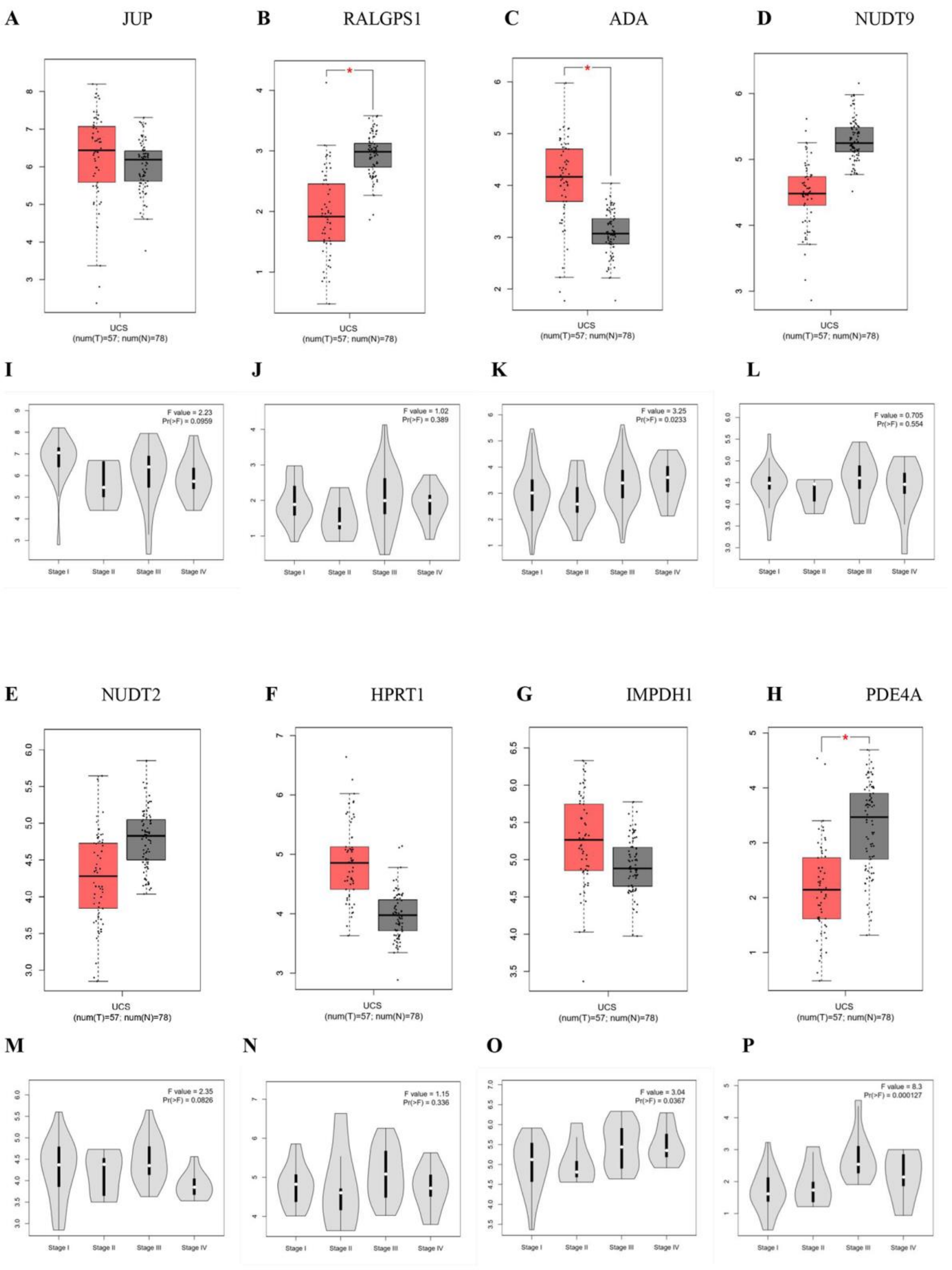

Figure S3 GEPIA database validation. RALGPS1 (B), NUDT9 (D), NUDT2 (E), and PDE4A (H) were significantly down regulated in tumor tissues. ADA (K), IMPDH1 (O), and PDE4A (P) were significantly correlated with clinical stage.

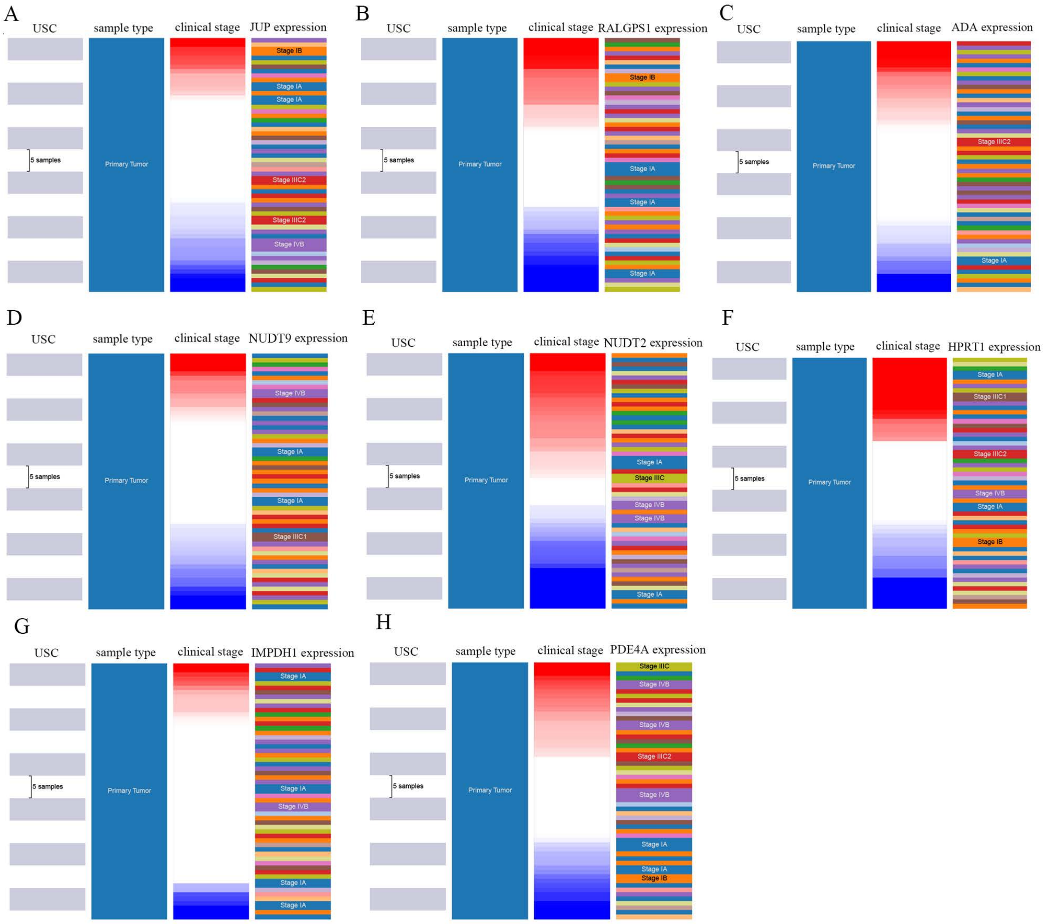

Figure S4 UCSC xena database validation. The expression levels of JUP (A), RALGPS1 (B), NUDT2 (E), HPRT1 (F), and IMPDH1 (G) were relatively high expressed in early stage UCS patients compared to advanced UCS patients. ADA (C), NUDT9 (D), and PDE4A (H) were elatively low expressed in early stage UCS patients compared to advanced UCS patients.

A

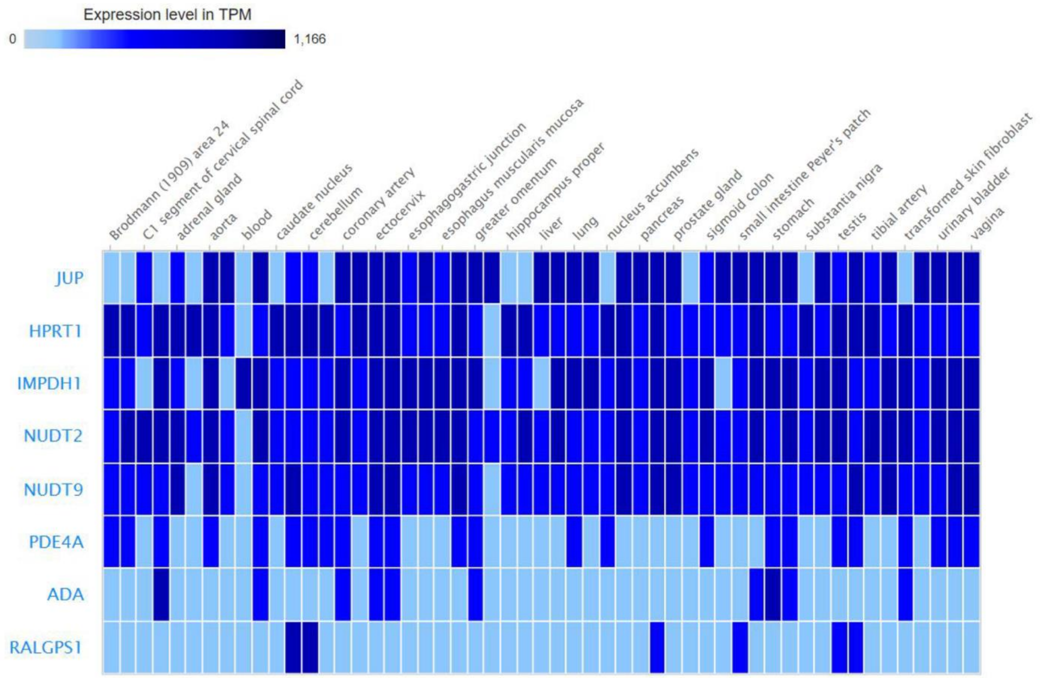

Figure S5 GTEx database validation. Heatmaps shown the expression levels of the genes in different normal tissues.

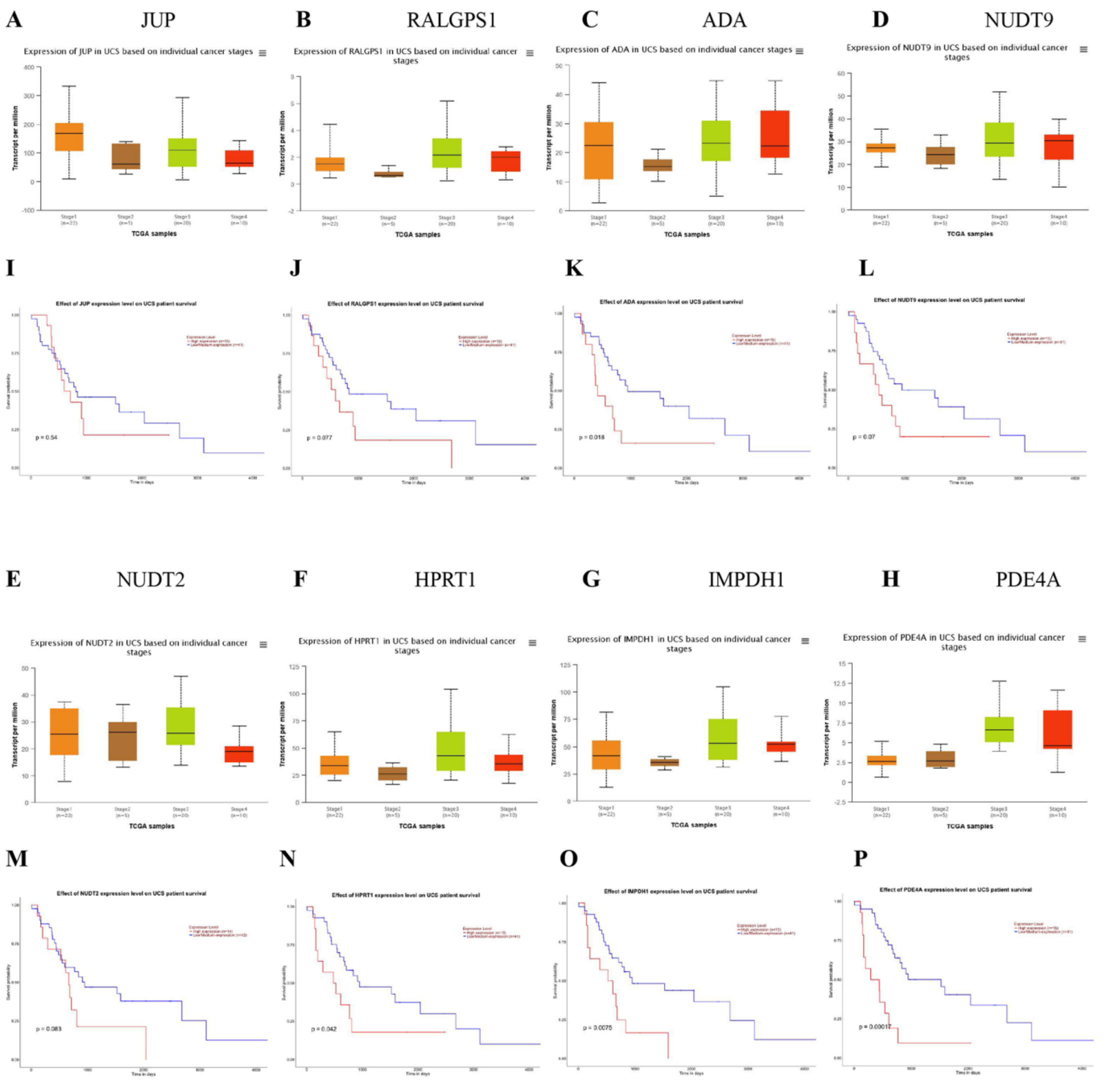

Figure S6 UALCAN database validation. JUP (A), ADA (C), NUDT2 (E), IMPDH1 (G), and PDE4A (H) were all differentially expressed between tumor and normal tissues with significance. Besides, ADA (K), HPRT1 (N), IMPDH1 (O), and PDE4A (P) were significantly correlated with patients’ prognosis.

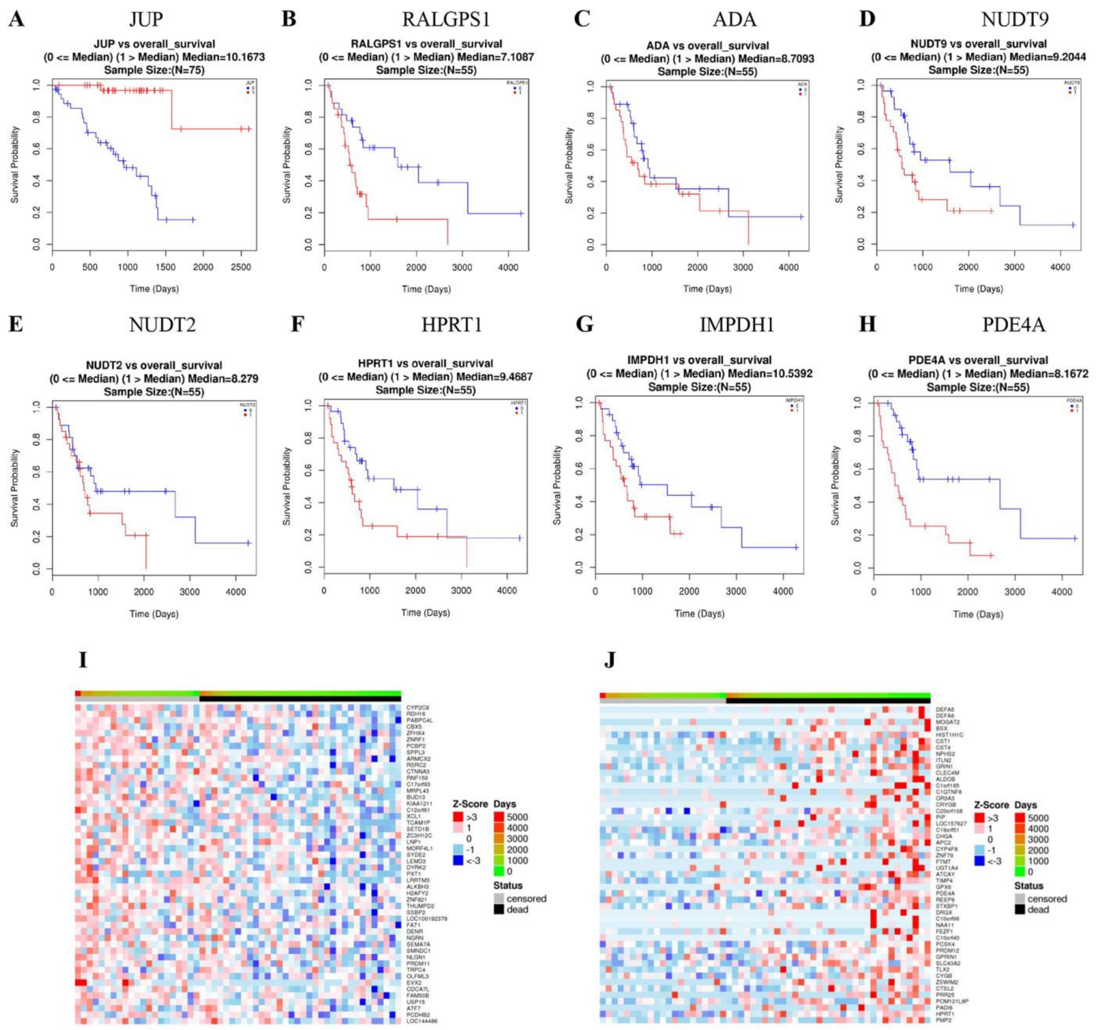

Figure S7 LinkedOmics database validation. K-M curves shown high expression of RALGPS1 (B), NUDT9 (D), HPRT1 (F), IMPDH1 (G), and PDE4A (H) were positively related to negative prognosis in patients with UCS. (I, J) Heatmaps shown the genes which have noteworthy correlation coefficient with RALGPS1.

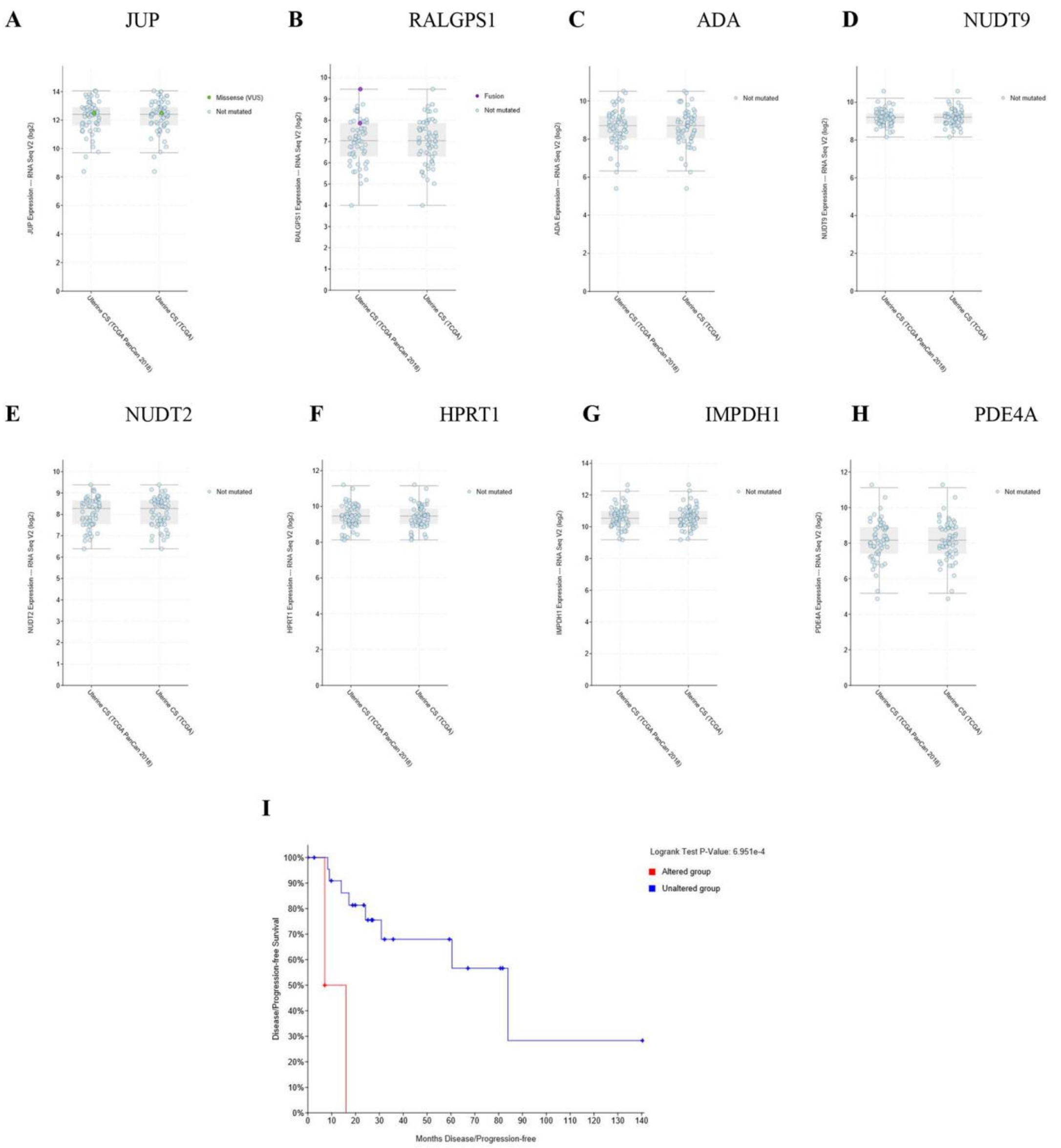

Figure S8 CBioportal database validation. Expression levels of JUP (A), RALGPS1 (B), ADA (C), NUDT9 (D), NUDT2 (E), HPRT1 (F), IMPDH1 (G), and PDE4A (H) in patients with UCS. (I) Integrated genes (P = 0.0007) were significantly related to prognosis.

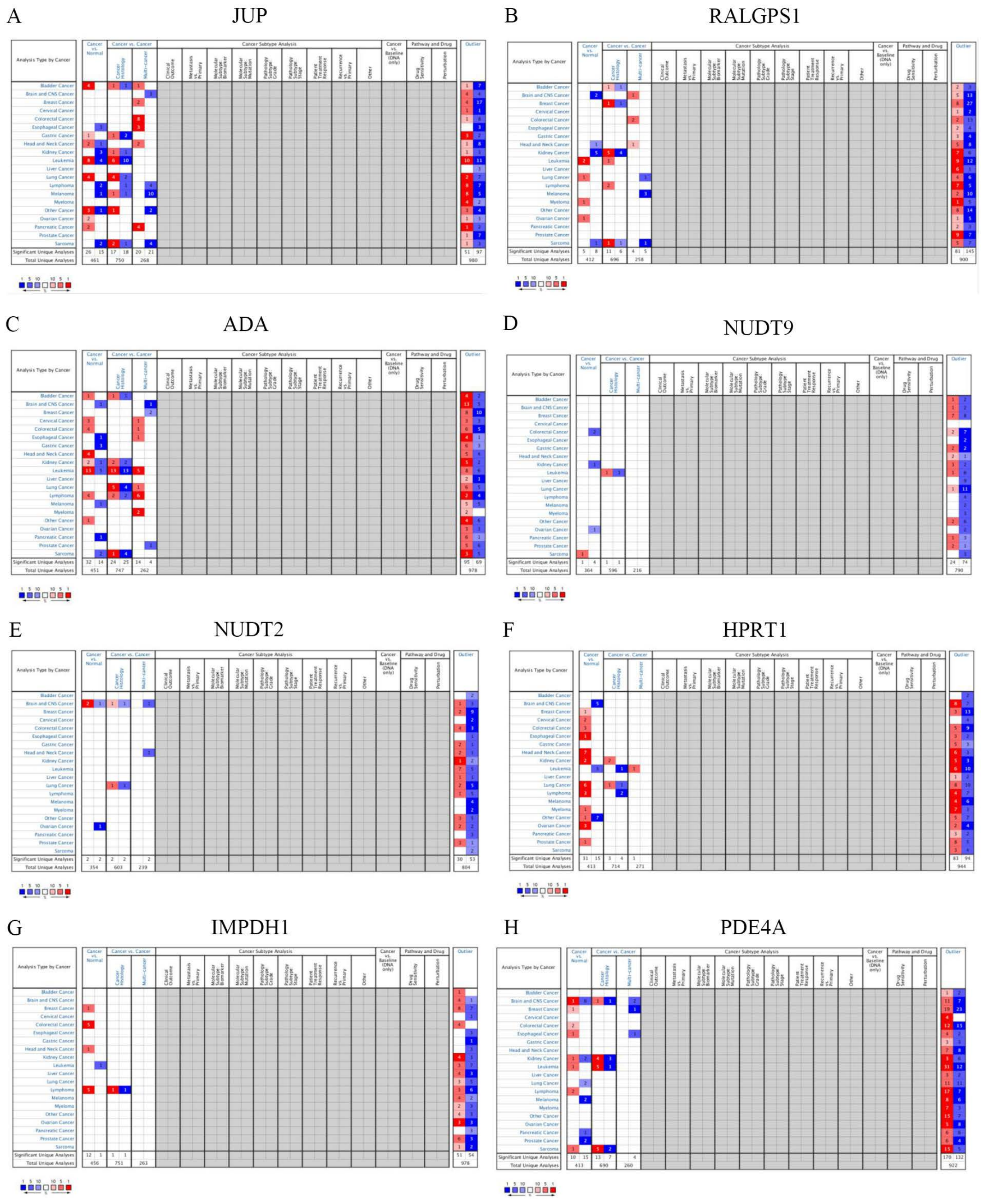

Figure S9 Oncomine database validation. JUP (A), ADA (C), HPRT1 (F), and IMPDH1 (G) were significantly up regulated, while RALGPS1 (B), NUDT9 (D), NUDT2 (E), and PDE4A (H) were down regulated in different kinds of uterine cancers.

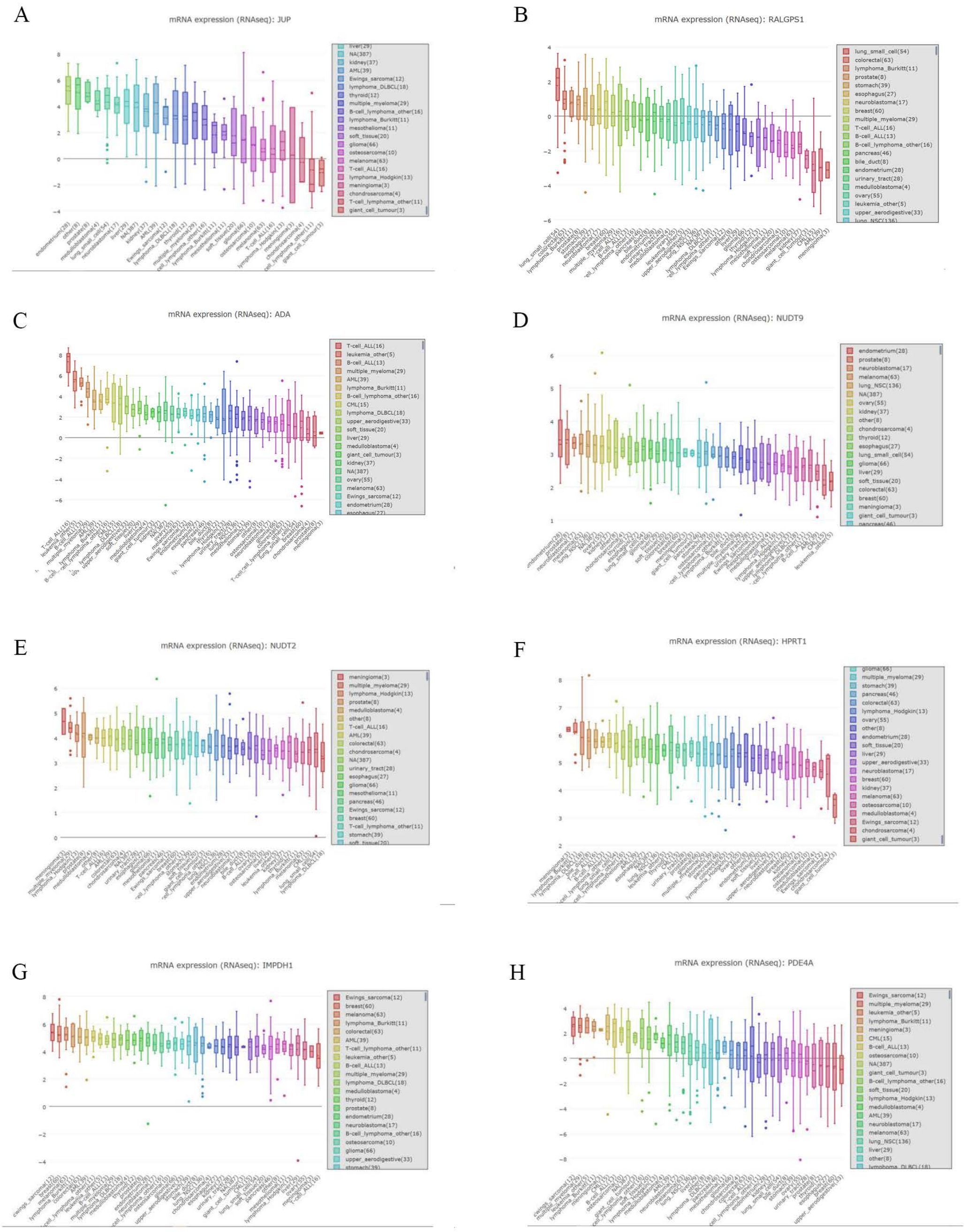

Figure S10 CCLE database validation. RALGPS1 (B), NUDT9 (D), NUDT2 (E), and PDE4A (H) were down regulated, while JUP (A), ADA (C), HPRT1 (F), and IMPDH1 (G) were significantly up regulated in different kinds of uterine cancers.

A

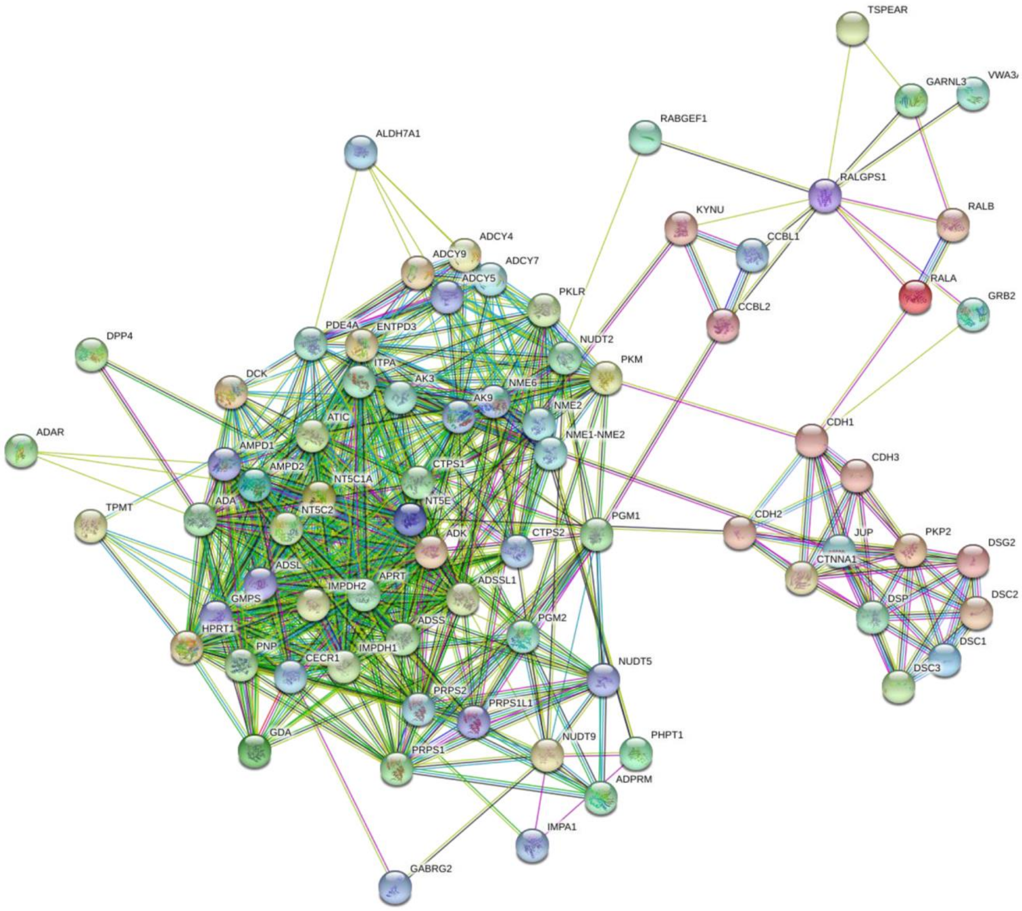

Figure S11 STRING database validation. Protein-protein interaction network shown JUP, RALGPS1, ADA, NUDT9, NUDT2, HPRT1, IMPDH1 and PDE4A were closely interacted.

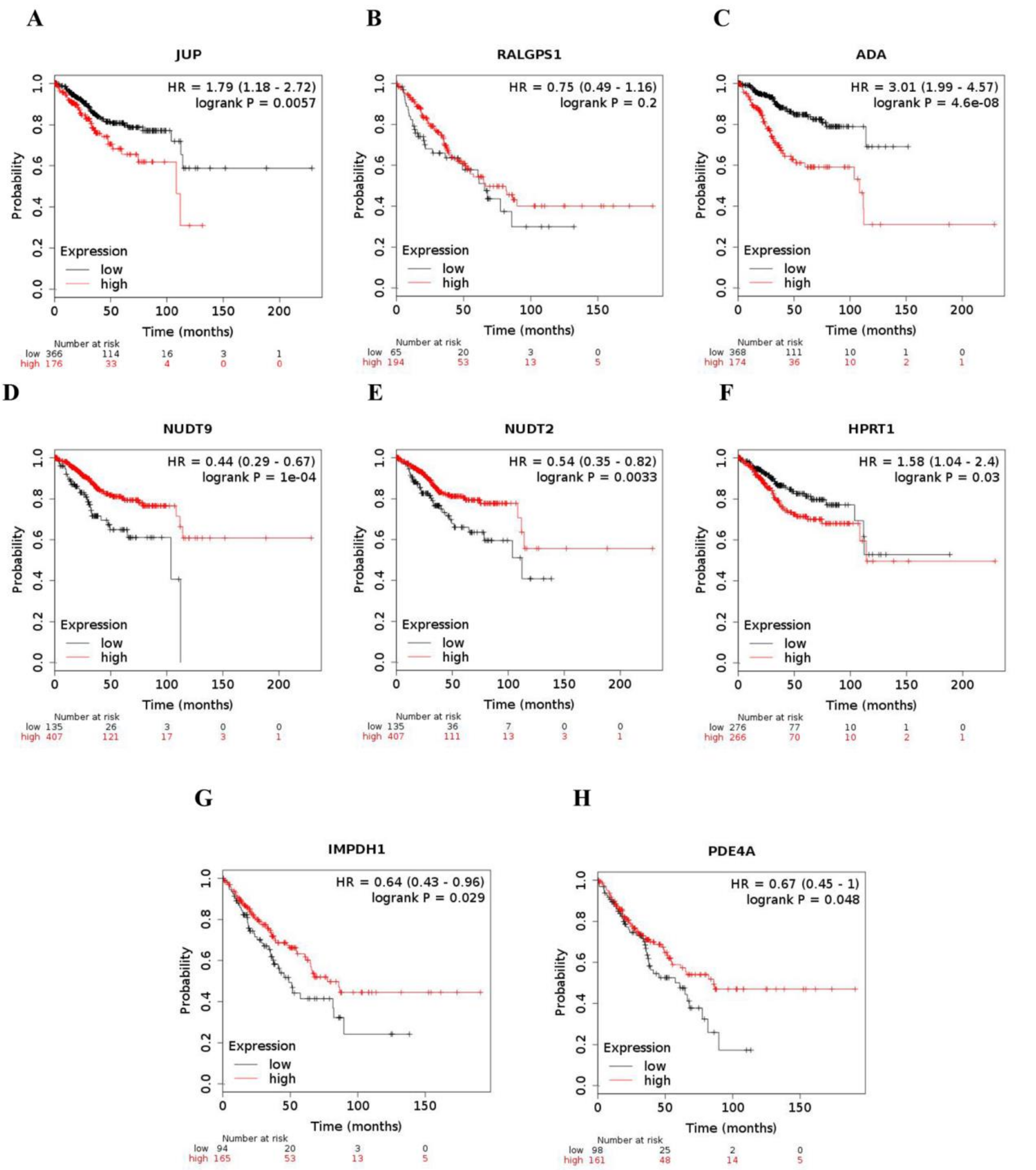

Figure S12 K-M Plotter database validation. JUP (A), ADA (C), NUDT9 (D), NUDT2 (E), HPRT1 (F), IMPDH1 (G), and PDE4A (H) were significantly correlated with patients' prognosis.
